# GBA1-dependent membrane glucosylceramide reprogramming promotes liver cancer metastasis via activation of the Wnt/β-catenin signalling pathway

**DOI:** 10.1038/s41419-022-04968-6

**Published:** 2022-05-30

**Authors:** Zhidong Qiu, Xuehong Wang, Zebin Yang, Sicong Liao, Wei Dong, Tian Sun, Huixian Wu, Qinqin Zhang, Zhixiong Pan, Sin Man Lam, Guanghou Shui, Junfei Jin

**Affiliations:** 1 Xiangya Hospital, Central South University, Changsha, 410008 Hunan China; 2 Guangxi Key Laboratory of Molecular Medicine in Liver Injury and Repair, the Affiliated Hospital of Guilin Medical University, Guilin, 541001 Guangxi China; 3 Department of General Surgery, Yantian District People’s Hospital, Shenzhen, 518081 Guangdong China; 4 Guangxi Health Commission Key Laboratory of Basic Research in Sphingolipid Metabolism Related Diseases, the Affiliated Hospital of Guilin Medical University, Guilin, 541001 Guangxi China; 5 Laboratory of Hepatobiliary and Pancreatic Surgery, the Affiliated Hospital of Guilin Medical University, Guilin, 541001 Guangxi China; 6 Department of Thyroid and Breast Surgery, Nanxishan Hospital of Guangxi Zhuang Autonomous Region, Guilin, 541002 China; 7 LipidALL Technologies Company Limited, Changzhou, China; 8 State Key Laboratory of Molecular and Developmental Biology, Institute of Genetics and Developmental Biology, Chinese Academy of Sciences, Beijing, China; 9 China-USA Lipids in Health and Disease Research Center, Guilin Medical University, Guilin, 541001 Guangxi China

**Keywords:** Cancer metabolism, Metastasis

## Abstract

The effect of glucosylceramide (GlcCer) reprogramming on liver cancer metastasis remains poorly understood. In this study, we demonstrated that the protein expression of GBA1, which catalyses the conversion of GlcCer to ceramide, was downregulated in liver cancer tissue. A clinical relevance analysis revealed that low expression of GBA1 was associated with the metastatic potential of liver cancer cells. Furthermore, loss- and gain-of-function studies confirmed that low expression of GBA1 promoted metastasis of liver cancer both in vitro and in vivo. Mechanistic studies indicated that low expression of GBA1 enhanced the metastatic ability of liver cancer by promoting the epithelial-mesenchymal transition (EMT), in which Wnt signalling pathway is involved. In the plasma membrane (PM), GBA1-dependent GlcCer reprogramming increased LRP6 location in the PM leading to an interaction between GlcCer and LRP6, subsequently promoting LRP6 phosphorylation at Ser1490, and finally activating the Wnt/β-catenin signalling pathway. To our knowledge, this is the first time to be found that GlcCer interacted with a protein. In addition, the results of mass spectrometry indicated that GlcCer d18:1/18:0 was the most notably increased studied species in the PM when GBA1 was downregulated, suggesting that GlcCer d18:1/18:0 may be the major functional lipid that promotes GBA1-dependent liver cancer metastasis. Thus, GBA1-mediated GlcCer reprogramming in the PM promotes metastasis of liver cancer via activation of the Wnt/β-catenin signalling pathway, upregulation of GBA1 may be a potential therapeutic strategy to combat liver cancer metastasis.

## Introduction

Liver cancer is the sixth most frequently diagnosed cancer worldwide and the third leading cause of cancer-related death. There were 906,000 new cases and 830,000 patients who died of liver cancer throughout the world in 2020 [1]. Metastasis is the major characteristic of liver cancer, and vascular invasion and extrahepatic tissue spread are known to lead to poor patient prognosis [2, 3]; however, the mechanisms of liver cancer cell invasion and metastasis remain poorly understood.

The epithelial-mesenchymal transition (EMT) plays an important role in the metastasis of cancer [4, 5]. It is a reversible programme that transforms epithelial cells into acquiring a mesenchymal cell phenotype. Once the EMT is activated, the polarity of epithelial cells is lost, and the action of epithelial cadherin (E-cadherin), which maintains adherens junctions between cells, is repressed. Similar to mesenchymal cells, epithelial cells can express neural cadherin (N-cadherin), vimentin and fibronectin, all of which are regulated by EMT-transcription factors, including Snail, Slug and ZEB1 [6]. Among the signalling pathways involved in EMT, the canonical Wnt pathway is particularly important [6, 7]. β-Catenin plays a critical role in the Wnt signalling pathway [8, 9]. When Wnt signalling is activated, a trimeric complex comprising Wnt (humans express 19 kinds of Wnt genes), the Wnt receptor Frizzled (FZD), and low-density lipoprotein receptor-related protein 5/6 (LRP5/6), is formed in the plasma membrane, followed by phosphorylation of LRP6 [9]. Then, the phosphorylation and degradation of β-catenin are prevented; subsequently, β-catenin accumulates in the cytoplasm and is translocated to the nucleus, where β-catenin regulates the expression of target genes such as c-myc and cyclin D1 [10].

Metastasis is a multi-step process known as the invasion-metastasis cascade [11, 12]. Metabolic reprogramming is involved in the multi-step metastatic cascade, and it involves glucose, nucleic acids and lipids [13, 14]. An increasing number of studies have demonstrated that abnormal metabolism, including sphingolipid (SPL) metabolism reprogramming, is associated with the EMT [14–17]. The SPL metabolism signalling pathway contains many bioactive lipid molecules, such as ceramide (Cer), glucosylceramide (GlcCer) and sphingomyelin [18, 19]. SPL is a necessary component of the eukaryotic cell plasma membrane [20], and glycosphingolipids (GSLs) generated from GlcCer are major SPLs in the plasma membrane, in addition to sphingomyelin. GlcCer is not only as a precursor of GSLs, including lactosylceramide (LacCer), ceramide trihexoside (Gb3) and monosialodihexosyl ganglioside (GM3), but is also a structural component of the plasma membrane. Previous studies demonstrated that the level of GlcCer is associated with the proliferation and chemoresistance of malignant tumours [21, 22], but the relationship between GlcCer metabolism reprogramming and metastasis in malignant tumours is still unclear.

Two types of enzymes, the synthetase glucosylceramide synthase (GCS) and catabolic enzymes, namely, GBA1, GBA2 and GBA3, are involved in GlcCer metabolism. On the one hand, ceramide serves as a substrate in the formation of GlcCer catalysed by GCS in the Golgi, and then, GlcCer is converted into complex GSLs that are transported to the plasma membrane. In addition, GlcCer can be directly transported to the plasma membrane by GLTP transport proteins [23, 24]. On the other hand, GlcCer is catabolized into ceramide and glucose mainly by GBA1 in lysosomes [18]. The mutation of GBA1 leading to Gaucher disease is associated with an increased incidence of cancer, and some evidence suggests that GBA1 is related to the pathogenesis of Parkinson’s disease [25–27]. Although mutation of GBA1 increases the incidence of cancer, including liver cancer, and one recent study showed that inhibition of GBA1 leads to reversal of gastric cancer chemoresistance [28], the role of GBA1 in cancer progression remains largely unknown.

Although some studies have demonstrated that alteration of lipid metabolism affects the activation of the Wnt signalling pathway [29, 30], the relationship between GlcCer and other GSLs with the Wnt signalling pathway remains poorly understood. In recent years, complementary biochemical and biophysical perspectives obtained through cancer studies have been proposed [31]. As important components of the plasma membrane, GlcCer and other GSLs are primarily enriched in lipid rafts in the plasma membrane [32]. Changes in the levels of GlcCer and/or other GSLs lead to the modification of lipid rafts, which are enriched with phospholipids, cholesterol and GSLs. Lipid rafts have been proven to be platforms for receptor signalling at the cell surface [33]. Therefore, we hypothesised that reprogramming of GlcCer in the plasma membrane regulates Wnt receptors on the cell surface and thus affects the activation of the Wnt signalling pathway. In this study we characterised the relationship between the metabolic reprogramming of GlcCer in the plasma membrane and the activation of the Wnt signalling pathway and discovered that GBA1 plays an important role in liver cancer metastasis, confirming that it is a critical enzyme that affects the reprogramming of GlcCer in the plasma membrane. Therefore, we also tested its potential therapeutic value in liver cancer metastasis.

## Results

### Low expression of GBA1 is associated with the metastatic potential of liver cancer cells

**Fig. 1:**
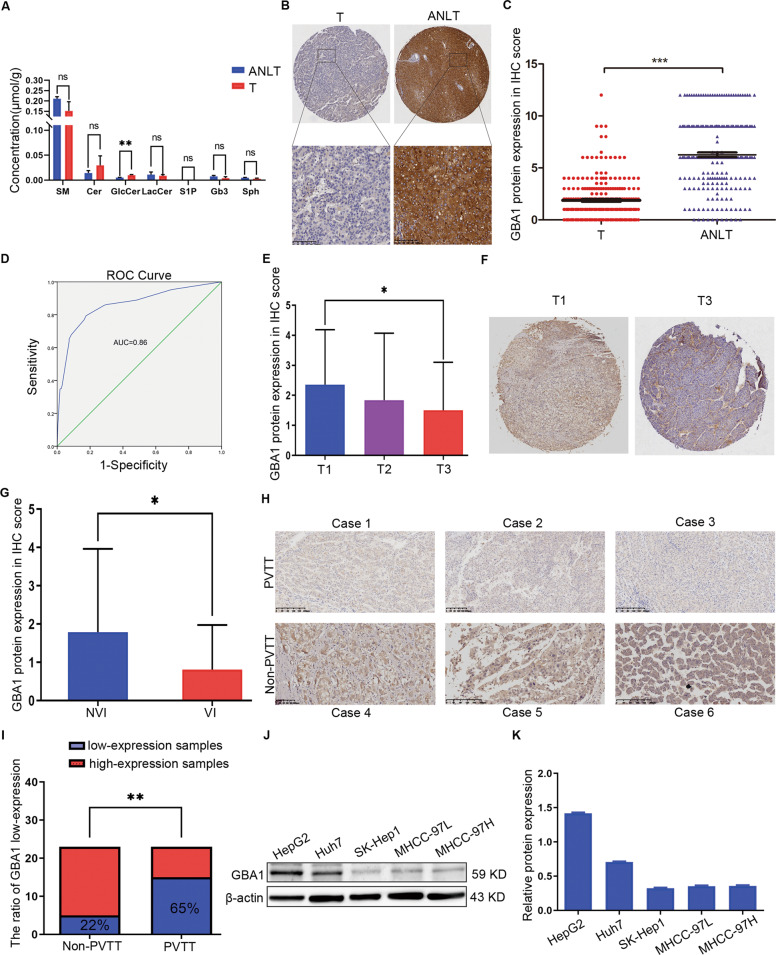
Low expression of GBA1 is associated with the metastatic potential of liver cancer cells. **A** Lipidomic analysis of sphingolipids in 3 pairs of liver cancer tissues (T) and the paired adjacent nontumorous liver tissues (ANLT). **B** Representative images of immunohistochemistry (IHC) staining of GBA1 in liver cancer tissues (T) and paired adjacent nontumorous liver tissue (ANLT). Scale bar, 100 μm. **C** Quantitative analysis of GBA1 protein levels in 233 paired samples of T and ANLT according to IHC scores. Statistical significance was analysed by Student’s *t* test. **D** ROC curve analyses show the diagnostic potential of GBA1 in liver cancer. **E** Quantitative analysis of GBA1 protein levels in T1 (*n* = 62), T2 (*n* = 121), and T3 (*n* = 46) stages according to IHC scores. Statistical significance was determined by one-way ANOVA, and LSD was performed after ANOVA to as a post hoc multiple comparison test. **F** Representative images of IHC stained GBA1 in tissues in the T1 and T3 stage. **G** Quantitative analysis of GBA1 protein levels in the nonvascular invasion (NVI, *n* = 125) and vascular invasion (VI, *n* = 21) groups according to IHC scores. **H** Representative images of IHC staining of GBA1 in the cases with PVTT and non-PVTT. **I** Real-time quantitative RT-PCR analysis of GBA1 mRNA in the non-PVTT (*n* = 33) and PVTT (*n* = 33) groups. Statistical significance was determined by chi-square test. The data are shown as the percentage of total specimens. **J**, **K** Western blot analysis of GBA1 protein in liver cancer cell lines with different metastatic potentials and relative GBA1 protein expression in different liver cancer cell lines. *p < 0.05, **p < 0.01, ***p < 0.001, ns, not significant.

In order to investigate the role of sphingolipids in liver cancer, a lipidomic analysis of sphingolipids was performed using liver cancer tissues and the paired adjacent nontumorous liver tissues. To our surprise, the data from three individual samples hinted the level of GlcCer was increased in the liver cancer tissues compared to the paired adjacent nontumorous liver tissues, but the other sphingolipids including Cer, sphingomyelin, LacCer, Gb3, sphingosine-1-phosphate and sphingosine did not change significantly (Fig. 1A). Because GlcCer was increased in liver cancer and GBA1 is an important catabolic enzyme of GlcCer, next we focused on GBA1. First, the expression of GBA1 at the protein level in liver cancer tissue and paired adjacent nontumorous liver tissue was determined by immunohistochemistry (IHC). We found that the protein level of GBA1 decreased significantly in liver cancer tissues compared to paired adjacent nontumorous liver tissue (Fig. 1B, C). GBA1 was mainly expressed in hepatocytes of the liver, and its subcellular localisation was found to be the cytoplasm (Fig. 1B). Second, a receiver operating characteristic curve (ROC curve) was generated according to the pathological score obtained from our IHC results, and the area under the curve was 86% (Fig. 1D). These results indicate that GBA1 is downregulated in liver cancer and that GBA1 may be a potential diagnostic marker for liver cancer.

To study the role of GBA1 in liver cancer, the clinical relevance of the expression of GBA1 in liver cancer was analysed. The level of GBA1 protein expression was associated with different T stages of liver cancer, GBA1 expression in stage T3 was lower than that in stage T1 (Fig. 1E, F); however, no significant difference in GBA1 expression was apparent between stages T1 and T2 or between stages T2 and T3 (Fig. 1E). Furthermore, the expression of GBA1 in liver cancer with vascular invasion was reduced compared to that without vascular invasion (Fig. 1G, H). Then, the mRNA expression of GBA1 in liver cancer tissues and adjacent nontumorous liver tissues was determined by real-time PCR, and we found that the ratio of low GBA1 expression was higher in patients with portal vein tumour thrombus (PVTT) than in those without PVTT (Fig. 1I). Next, we analysed the expression of GBA1 in several liver cancer cell lines with differing metastatic potential, and the results showed that the expression of GBA1 was profoundly decreased in MHCC-97H, MHCC-97L and SK-Hep1 with high metastatic potential compared with that in HepG2 and Huh7 with lower metastatic potential (Fig. 1J, K). These data suggested that low expression of GBA1 is associated with the metastatic potential of liver cancer cells.

### GBA1 deficiency promotes metastasis of liver cancer in vitro and in vivo

**Fig. 2:**
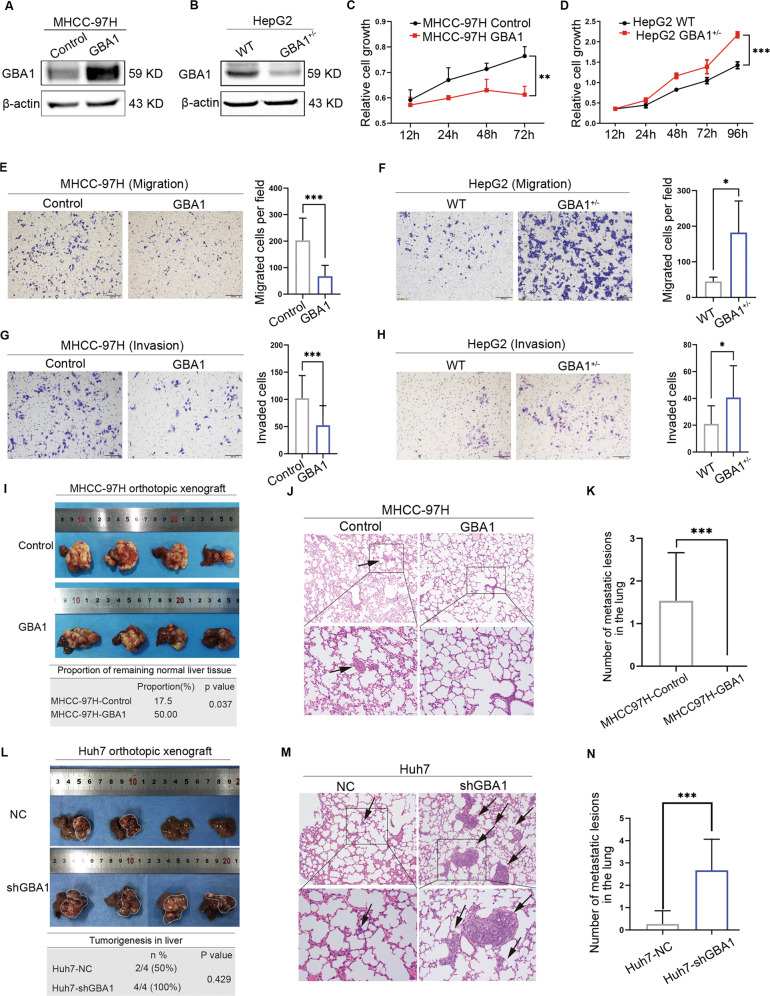
Downregulation of GBA1 promotes metastasis of liver cancer in vitro and in vivo. **A**, **B** Confirmation of GBA1 overexpression in MHCC-97H cells and knockdown in HepG2 liver cancer cell lines. **C**, **D** The effects of GBA1 gain- or loss-of-function on the proliferation of MHCC-97H and HepG2 cells in vitro. The relative cell growth is shown as an OD value. **E**, **G** The effect of GBA1 gain-of-function on the migration and invasion of MHCC-97H cells. Representative images of the cells described in **E** (left) and **G** (left), and the relative number of migrated and invaded cells are shown as the number of migrated cells per field in **E** (right) and invaded cells in **G** (right). **F**, **H** The effect of GBA1 loss-of-function on the migration and invasion of HepG2 cells. Representative images are shown in **F** (left) and **H** (left), and the relative number of migrated and invaded cell numbers is shown as the number of migrated cells per field in **F** (right) and invaded cells in **H** (right). **I** The effect of GBA1 gain-of-function on tumour volume in the orthotopic xenograft model (top). The data are shown as the means ± SD (*n* = 4). The effect of GBA1 gain-of-function on the proportion of normal liver tissue in the orthotopic xenograft model. The data are shown as the percentage of normal liver tissue compared with the whole liver after treatment (down, *n* = 4). Significance was determined by chi-square test. **J**, **K** The effect of GBA1 gain-of-function on spontaneous lung metastasis in the orthotopic xenograft model. Representative images of lung metastatic lesions as indicate by H&E staining are shown (**J**), and the average number of lung metastatic lesions in the four mice in each group is shown as the means ± SD (**K**). Significance was determined by Student’s *t* test. The arrow indicates the metastatic lung lesions. **L** The effect of GBA1 loss-of-function on the tumour volume in the orthotopic xenograft model (top) and the effect of GBA1 loss-of-function on liver cancer formation in the orthotopic xenograft model. The data are shown as the percentage of liver with cancer (bottom) (*n* = 4). Significance was determined by Fisher’s exact probability test. **M**, **N** The effect of GBA1 loss-of-function on spontaneous lung metastasis in the orthotopic xenograft model. Representative images of metastatic lung lesions as indicated by haematoxylin and eosin (H&E) staining are shown (**M**), and the average number of metastatic lung lesions in the four mice in each group is shown as the mean ± SD (**N**). Significance was determined by Student’s *t* test, and arrows indicate metastatic lung lesions. *p < 0.05, ***p < 0.001, ***p < 0.001; ns, not significant. Scale bar, 50 μm (upper images in **J** and **M**), Scale bar, 100 μm (lower images in **J**, **M**).

To further study the functional role of GBA1 in liver cancer, we performed gain- and loss-of-function studies in vitro. First, we upregulated the expression of GBA1 in MHCC-97H cells with lower GBA1 expression (Fig. 2A). In addition, we tried to knock out GBA1 by CRISPR-Cas9 in HepG2 cells with high GBA1 expression, but we only successfully obtained heterozygous HepG2 cells with GBA1 knockdown (Fig. 2B). Then, proliferation, migration and invasion experiments were performed with the aforementioned MHCC-97H cells and with HepG2 cells. Overexpression of GBA1 reduced the MHCC-97H cell proliferation rate (Fig. 2C); in contrast, the proliferation of HepG2 cells was increased when GBA1 was knocked down (Fig. 2D). Consistent with these findings, the migration and invasion of MHCC-97H cells were inhibited when GBA1 was upregulated, and the migratory and invasive abilities of HepG2 cells were obviously increased when GBA1 was knocked down (Fig. 2E–H). These data indicated that low expression of GBA1 promotes metastasis of liver cancer and that GBA1 may play an important role in suppressing the metastasis of liver cancer.

To confirm that GBA1 suppresses the metastasis of liver cancer, we examined the effect of GBA1 deficiency on the growth and metastasis of liver cancer using an orthotopic xenograft model. First, MHCC-97H cells overexpressing GBA1 and Huh7 cells deficient in GBA1 were injected into the left lobe of the liver in nude mice to establish orthotopic xenografts. The mice were sacrificed 6 weeks after injection, and the primary tumours and lungs were collected. Then, the tumour volume, the proportion of normal liver tissues, and the number of metastatic lesions in the lung were analysed. The results showed that overexpression of GBA1 appeared to reduce tumour growth, but no significant difference in tumour volume was observed between the two groups (Figs. 2I and S1A). However, the proportion of normal liver tissue was greater in the mice in which liver cancer had been established with MHCC-97H cells overexpressing GBA1 (Fig. 2I), indicating that overexpression of GBA1 may help preserve normal liver tissue and prevent liver cancer invasion. Furthermore, no metastatic lesions in the lung were observed in the mice with liver cancer that had been established by MHCC-97H cells overexpressing GBA1, indicating that GBA1 overexpression suppressed the metastasis of liver cancer to the lung (Fig. 2J, K). Interestingly, in the control group, one metastatic lesion was observed in a vessel located at the junction between tumour tissue and normal liver tissue, but no metastatic lesion was found in the GBA1-overexpressing group, suggesting that GBA1 overexpression suppressed vascular metastasis of liver cancer (Fig. S1B and C). In contrast, the tumour volume in mice injected with GBA1-silent Huh7 cells was higher than that in the control group (Figs. 2L and S1D). The percentage of livers with cancer formation was higher in the mice injected with GBA1-silenced Huh7 cells than in the mice injected with control Huh7 cells (Fig. 2L). As expected, the number of metastatic lesions in the lung was remarkably increased in the mice injected with GBA1-silent Huh7 cells (Fig. 2M, N), indicating that Huh7 cells exhibit a higher potential for metastasis upon downregulation of GBA1. Similar results were observed when we used the HepG2 cell line with GBA1 knocked down to establish orthotopic xenograft models and compared the results with those obtained for the Huh7 cell line (Fig. S2A–D). Taken together, this gain- and loss-of-function study in vivo confirmed that GBA1 inhibition promotes metastasis of liver cancer.

### Low expression of GBA1 promotes the epithelial-mesenchymal transition (EMT) via activation of the Wnt/β-catenin signalling pathway

**Fig. 3:**
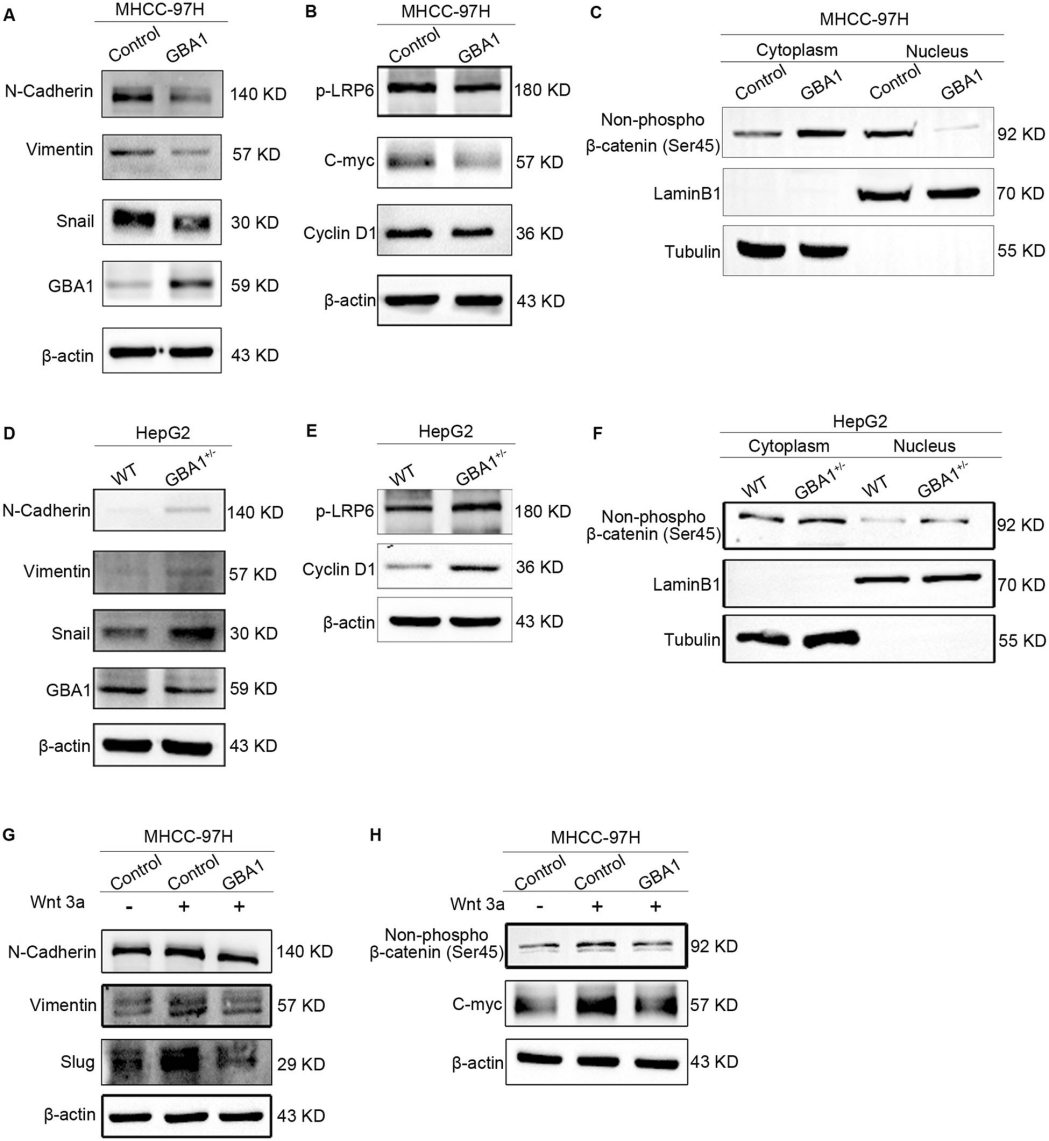
Low expression of GBA1 promotes the epithelial-mesenchymal transition (EMT) via activation of the Wnt/β-catenin signalling pathway. **A**, **D** The expression of EMT markers in MHCC-97H cells with stable GBA1 overexpression (**A**) or HepG2 cells with stable GBA1 downregulation (**D**) as detected by Western blotting. **B**, **E** The expression of phospho-LRP6 and the Wnt target genes c-myc and/or cyclin D1, which are related to the Wnt signalling pathway in MHCC-97H cells with stable GBA1 overexpression (**B**) or HepG2 cells with stable GBA1 downregulation (**E**) was detected by Western blotting. **C**, **F** Western blot analysis was performed to determine the level of non-phospho-β-catenin in the cytoplasm and nucleus of MHCC-97H cells with stable GBA1 overexpression (**C**) or HepG2 cells with stable GBA1 downregulation (**F**). **G** Western blotting was performed to analyse the effect of GBA1 overexpression on the Wnt3a-activating EMT. **H** Western blotting was performed to analyse the effect of GBA1 overexpression on the Wnt3a-activated Wnt signalling pathway.

As previously reported, the EMT is crucial for the invasion and metastasis of malignant cells [6]; therefore, in this study, we evaluated EMT marker expression in MHCC-97H cells with overexpression of GBA1 or in HepG2 cells with knockdown of GBA1 by performing Western blot analysis. The expression of markers of the mesenchymal cell state, namely, N-cadherin, vimentin and snail, was reduced when GBA1 was upregulated in MHCC-97H cells (Fig. 3A); in contrast, the expression of N-cadherin, vimentin and snail was increased after GBA1 was knocked down in HepG2 cells (Fig. 3D). These data demonstrated that low expression of GBA1 accelerates liver cancer metastasis by promoting EMT.

The Wnt signalling pathway is involved in the EMT. One study showed that the level of glucosylceramide may influence the catenin signalling pathway [34]. Given that GBA1 is an enzyme in glucosylceramide catabolism, we hypothesised that low expression of GBA1 in liver cancer promotes the EMT via activation of the Wnt signalling pathway. To evaluate this hypothesis, we first investigated a series of proteins related to the Wnt signalling pathway, such as phospho-LRP6, and target genes, such as c-myc and cyclin D1. The expression of phospho-LRP6, c-myc and cyclin D1 was decreased while that of GBA1 was upregulated in MHCC-97H cells (Fig. 3B), and the opposite results were obtained for HepG2 cells with GBA1 knockdown (Fig. 3E). Furthermore, we detected the protein level of non-phospho-β-catenin in the cytoplasm and nucleus because non-phospho-β-catenin in the nucleus plays a crucial role in the Wnt signalling pathway. The data showed that the level of non-phospho-β-catenin was decreased significantly in the nucleus but increased in the cytoplasm of MHCC-97H cells overexpressing GBA1 (Fig. 3C). However, the level of non-phospho-β-catenin was increased in both the nucleus and cytoplasm of HepG2 cells after knockdown of GBA1 (Fig. 3F). Next, to confirm that the low expression of GBA1 promoted the EMT was caused by activation of the Wnt signalling pathway, we observed the effect of GBA1 on Wnt3a activation of the Wnt signalling pathway. Overexpression of GBA1 effectively reduced the levels of N-cadherin, vimentin, snail, and slug, which had been increased by Wnt3a in MHCC-97H cells (Fig. 3G), and similar changes in non-phospho-β-catenin and c-myc were observed (Fig. 3H). We also performed an animal study to further confirm that the low expression of GBA1 promoted EMT was caused by activation of the Wnt signalling pathway. The IHC results showed that Wnt3a overexpression upregulated the proteins level of non-phospho-β-catenin, c-myc, N-cadherin, and vimentin in the tissue from the orthotopic xenograft of MHCC-97H cells, however, GBA1 overexpression could inhibit the increase of all these proteins caused by Wnt3a overexpression (Fig. S3A and B), Taken together, these results suggested that low expression of GBA1 promotes the EMT via activation of the Wnt/β-catenin signalling pathway.

### GBA1 regulates GlcCer levels in whole liver cancer cells and the plasma membrane of liver cancer cells

**Fig. 4:**
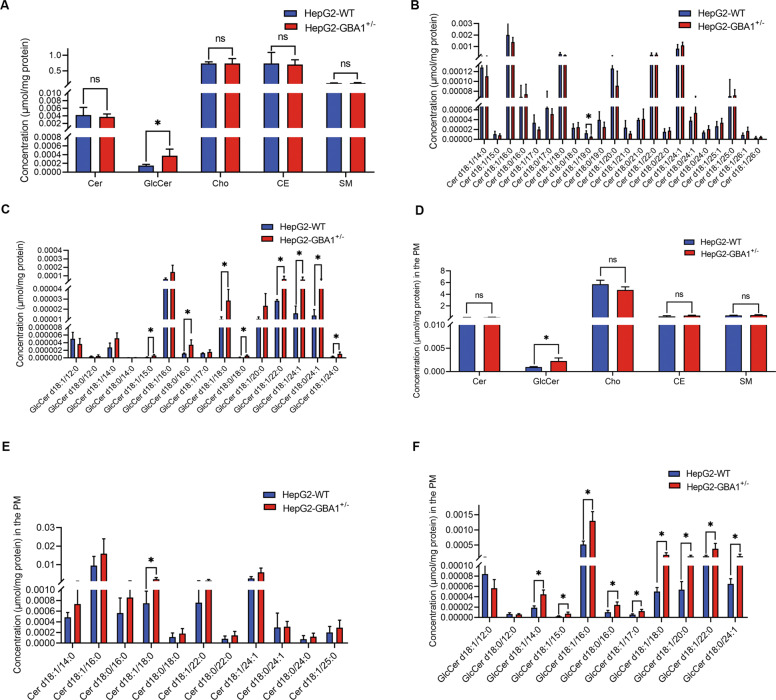
GBA1 regulates the GlcCer levels in whole liver cancer cells and the plasma membrane of liver cancer cells. **A** Total levels of GlcCer, Cer, cholesterol, cholesteryl esters, and sphingomyelin in HepG2 cells with stable GBA1 downregulation (*n* = 4/WT group, *n* = 4/downregulated group) were measured by mass spectrometry. Data are shown as concentrations (μmol/mg protein), and significance was determined by Student’s *t* test. **B**, **C** Levels of subspecies of Cer and GlcCer in HepG2 cells with stable GBA1 downregulation (*n* = 4/WT group, *n* = 4/downregulated group) were analysed by mass spectrometry. The data are shown as concentrations (μmol/mg protein), and significance was determined by Student’s *t* test. **D** Total levels of GlcCer, Cer, cholesterol, cholesteryl esters, and sphingomyelin in the plasma membrane of HepG2 cells with stable GBA1 downregulation (*n* = 4/WT group, *n* = 4/downregulated group) were measured by mass spectrometry. The data are shown as concentrations (μmol/mg protein), and significance was determined by Student’s *t* test. **E**, **F** Levels of subspecies of Cer and GlcCer in the plasma membrane of HepG2 cells with stable GBA1 downregulation (*n* = 4/WT group, n = 4/downregulated group) were analysed by mass spectrometry. The data are shown as concentrations (μmol/mg protein), and significance was determined by Student’s *t* test. *p < 0.05; ns, not significant.

Because GBA1 is an enzyme of GlcCer catabolism, we measured the levels of GlcCer by mass spectrometry. The cellular level of GlcCer was significantly increased after knockdown of GBA1 in HepG2 cells (Fig. 4A). We also measured the levels of Cer, cholesterol, cholesteryl esters and sphingomyelin, which are components of the plasma membrane and are involved in sphingolipid metabolism. The results showed that knockdown of GBA1 did not affect the levels of Cer, cholesterol, cholesteryl esters, or sphingomyelin (Fig. 4A). The cellular levels of Cer and GlcCer subspecies showed that only the level of Cer d18:0/19:0 was decreased when GBA1 expression was inhibited in HepG2 cells (Fig. 4B). As expected, when GBA1 expression was inhibited, most of the GlcCer subspecies were increased, including GlcCer d18:1/15:0, GlcCer d18:0/16:0, GlcCer d18:1/18:0, GlcCer d18:0/18:0, GlcCer d18:1/22:0, GlcCer d18:1/24:1, GlcCer d18:0/24:1 and GlcCer d18:1/24:0 (Fig. 4C). Taken together, these results demonstrated that low expression of GBA1 increased the level of cellular GlcCer.

Furthermore, we detected the levels of GlcCer and its subspecies in the plasma membrane because GlcCer is an important component of the plasma membrane. We isolated plasma membrane fractions from whole cells and then performed mass spectrometry to determine the levels of the aforementioned sphingolipids in the plasma membrane. Consistent with the cellular results described above, knockdown of GBA1 led to an increase in GlcCer levels in the plasma membrane of HepG2 cells, (Fig. 4D). Knockdown of GBA1 had no effect on the levels of Cer, cholesterol, cholesteryl esters, or sphingomyelin in the plasma membrane of HepG2 cells (Fig. 4D). The results of Cer subspecies in the plasma membrane showed that depletion of GBA1 increased only the level of Cer d18:1/18:0 among all the detected Cer subspecies (Fig. 4E). Although most of the major GlcCer subspecies were increased in the plasma membrane after knockdown of GBA1 (Fig. 4F), some subspecies, including GlcCer d18:0/14:0, GlcCer d18:0/18:0, GlcCer d18:1/24:1 and GlcCer d18:1/24:0, could not be detected in the plasma membrane. Interestingly, after knockdown of GBA1, the levels of some subspecies of GlcCer, including GlcCer d18:1/14:0, GlcCer d18:1/16:0 and GlcCer d18:1/17:0, increased in the plasma membrane but exhibited no change in total cells; other subspecies of GlcCer followed the same trends of change in the plasma membrane and total cells after GBA1 expression inhibition (Fig. 4F). These results indicated that low expression of GBA1 increased the level of GlcCer, especially in the plasma membrane. Among the GlcCer species, the level of GlcCer d18:1/18:0 was the most notably changed (Fig. 4F).

### Low expression of GBA1 increases the location of LRP6 in the cell plasma membrane and leads to an interaction between GlcCer and LRP6

**Fig. 5:**
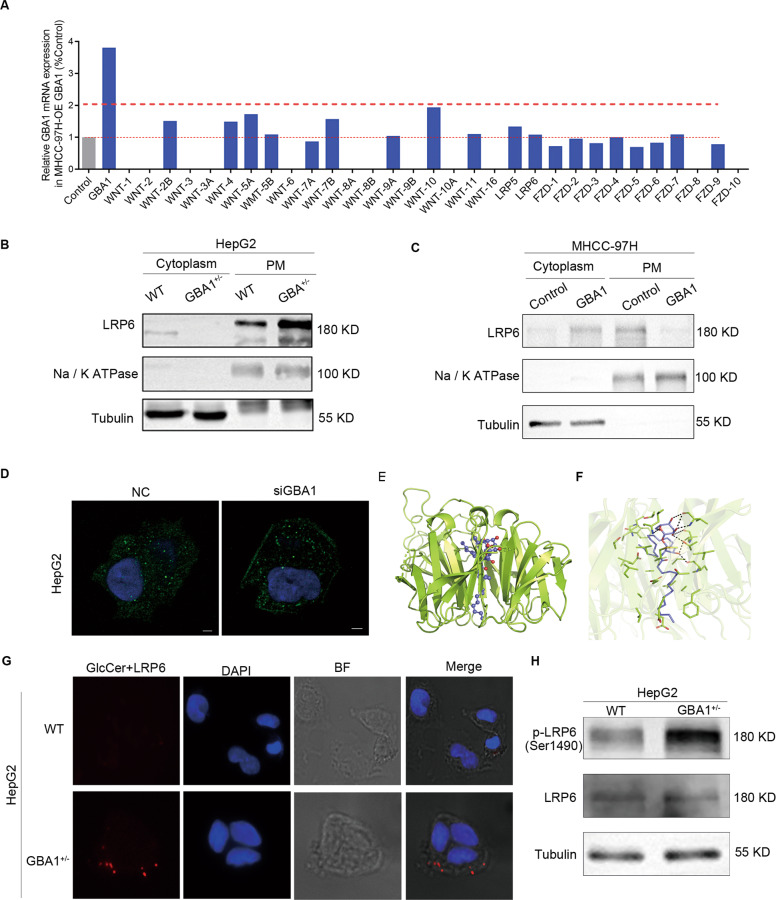
Low expression of GBA1 increases the levels of LRP6 in the cell plasma membrane and led to an interaction between GlcCer and LRP6. **A** Real-time quantitative RT-PCR analysis of Wnt molecules and Wnt receptors, including FZD1-10 and LRP5/6 mRNA, in MHCC-97H cells with stable GBA1 overexpression. **B**, **C** Western blotting was performed to determine the levels of LRP6 in the cytoplasm and plasma membrane in HepG2 cells with stable GBA1 downregulation and in MHCC-97H cells with stable GBA1 overexpression. **D** Immunofluorescence staining was used to detect the location of LRP6 in the plasma membrane after GBA1 was knockdown in HepG2 cells. scale bar, 10 μm. **E**, **F** Schematic representation of the binding mode of GlcCer and YWTD-EGF Domain Pair (PDB code: 1IJQ) of LRP6, Autodock was employed for docking and PyMOL was used for graph preparation. **G** An interaction of endogenous GlcCer and LRP6 was detected by PLA in HepG2 cells with stable GBA1 downregulation. BF: bright field. **H** Western blotting was performed for the phosphorylation of LRP6 at Ser1490 in HepG2 cells with stable GBA1 downregulation.

Inhibition of GBA1 led to an increase of GlcCer in the plasma membrane. As important components of the plasma membrane, GlcCer at changed levels may have effects on membrane receptors. After overexpression of GBA1 in MHCC-97H cells, we detected the mRNA expression of Wnt molecules and Wnt receptors, including FZD1-10 and LRP5/6. Interestingly, the mRNA levels of FZD1-10, LRP5/6, and all the tested Wnt molecules did not change significantly after overexpression of GBA1 (Fig. 5A). Next, the co-receptor LRP6 in the plasma membrane was detected by Western blotting because the phosphorylation of LRP6 plays a crucial role in the Wnt signalling [35]. The location of LRP6 was increased in the plasma membrane and reduced in the cytoplasm upon inhibition of GBA1 in HepG2 cells (Fig. 5B), In contrast, the protein level of LRP6 was decreased in the plasma membrane but increased in the cytoplasm after overexpression of GBA1 in MHCC-97H cells (Fig. 5C). In addition, we also studied the location of LRP6 in the plasma membrane in HepG2 cells with GBA1 silence by immunofluorescence. The result showed that when GBA1 was silent, LRP6 was obviously increased in the plasma membrane and decreased in the cytoplasm (Fig. 5D). Because ceramide could interact with P53 [36], we hypothesised that GlcCer increase in the plasma membrane may lead an interaction between GlcCer and LRP6. Firstly, we performed a modelling study for the binding between GlcCer and LRP6. YWTD-EGF Domain is an important extracellular domain of LRP6, GlcCer was docked into the binding pocket of the crystal structure of YWTD-EGF Domain Pair (PDB code: 1IJQ) [37], the putative binding mode rationalises the binding of GlcCer and LRP6 (Fig. 5E, F). Then, we confirmed the interaction between endogenous GlcCer and LRP6 in situ by proximity ligation assay (PLA) which is a more sensitive method than Co-IP to study two molecules’ interaction [38]. As expected, we found that GlcCer interacted with LRP6 after the knockdown of GBA1 (Fig. 5G). Moreover, deficiency of GBA1 in HepG2 cells increased LRP6 phosphorylation at Ser1490 (Fig. 5H). Taken together, these results demonstrated that low expression of GBA1 increased LRP6 location in the cell plasma membrane and led to an interaction between GlcCer and LRP6 subsequently promoting the phosphorylation of LRP6 at Ser1490. Until now, to our knowledge, this is the first time to be found that GlcCer interacted with protein.

### GlcCer plays a crucial role in promoting the metastasis of liver cancer

**Fig. 6:**
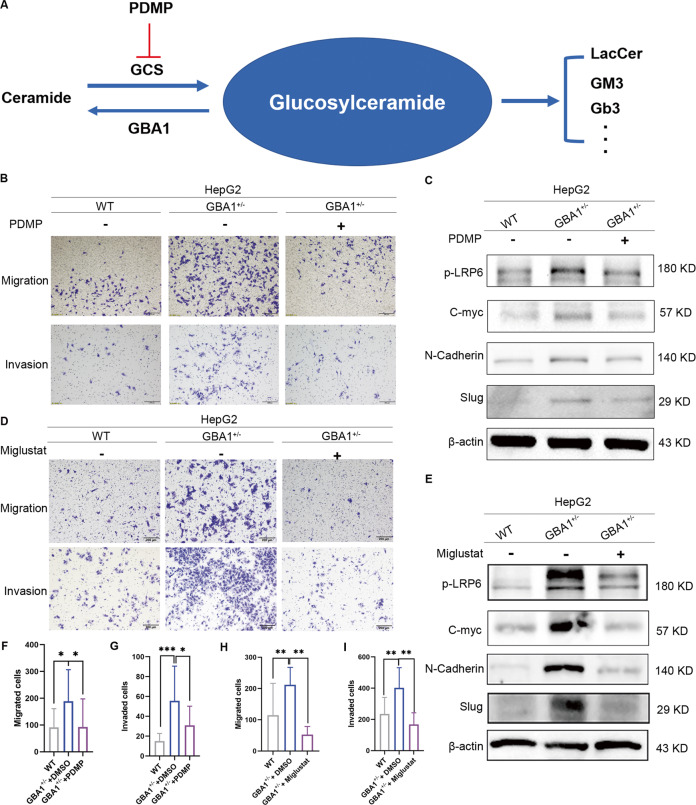
GlcCer plays a crucial role in promoting the metastasis of liver cancer cells. **A** Diagram of cellular GlcCer metabolism. **B**, **D** The effect of PDMP or miglustat on the mechanism of inhibited migration and invasion of HepG2 cells with GBA1 knockdown, scale bar, 200 μm. **C**, **E** Western blotting was performed to determine the effect of PDMP or miglustat on GBA1 deficiency-upregulated proteins involved in the Wnt signalling pathway and EMT. **F**–**I** The relative number of migrated cells (**F**, **H**) and invaded cells (**G**, **I**) is shown.

To confirm that GBA1 inhibition-promoting metastasis of liver cancer is mediated by GlcCer, we treated cells with PDMP, an inhibitor of GCS that synthesises GlcCer from Cer (Fig. 6A), to observe the effect of GlcCer changes on the inhibitory effect on the migration and invasion of HepG2 cells with GBA1 knockdown. Firstly, we performed a GCS activity assay in the HepG2 cells with GBA1 knockdown after the treatment of PDMP and found that PDMP could inhibit the activity of GCS effectively (Fig. S4A). In consistent with this result, the data from mass spectrometry also confirmed that PDMP could reduce the level of GlcCer in the HepG2 cells with GBA1 knockdown (Fig. S4B), As expected, PDMP treatment efficiently reversed the increase in migration and invasion of HepG2 cells induced by GBA1 deficiency (Fig. 6B, F, G). We also observed that PDMP affected the GBA1-dependent Wnt signalling pathway and EMT activation. As expected, PDMP reversed the increase in phospho-LRP6, c-myc, N-cadherin, and slug expressions in HepG2 cells that had been induced by GBA1 deficiency (Fig. 6C). In addition to PDMP, miglustat which is another inhibitor of GCS has a similar effect on Wnt activation and metastasis (Fig. 6D, E, H, I), These results demonstrated that GBA1 inhibition promotes metastasis of liver cancer in which GlcCer is involved.

### The GBA1-mediated increase in GlcCer level is associated with the metastasis of human liver cancer cells

**Fig. 7:**
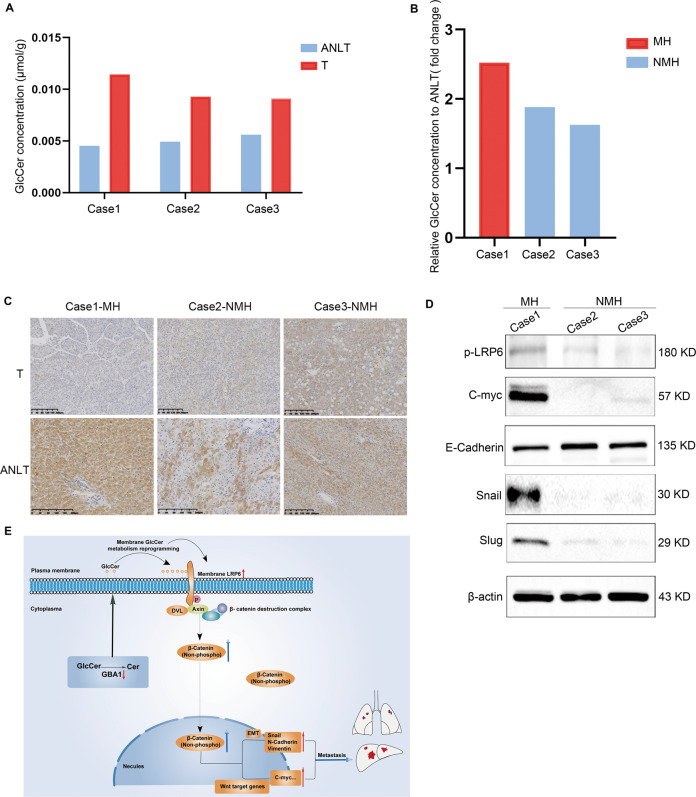
Downregulation of GBA1 is correlated with a high level of GlcCer, activation of the Wnt/β-catenin signalling pathway, and a high capacity for human liver cancer metastasis. **A** The levels of GlcCer in human liver cancer tissues (T, *n* = 3) and paired adjacent nontumorous liver tissue (ANLT, *n* = 3). The data on every pair of liver cancer tissues are shown as concentrations of GlcCer. **B** The level of GlcCer in human liver cancers with metastatic lesions (MHs) and nonmetastatic lesions (NMHs). The data are shown as the concentration of GlcCer. **C** The expression of GBA1 protein in human liver cancers (T) with metastatic lesions (case 1-MH) and no-metastatic lesions (case 2-NMH, case 3-NMH) and paired adjacent nontumorous liver tissue (ANLT), scale bar, 100 μm. **D** Western blotting was performed to determine the expression of Wnt signalling proteins and EMT markers in human liver cancers with metastatic lesions (MHs) and nonmetastatic lesions (NMHs). **E** Schematic depiction of the underlying mechanisms of low GBA1 promotes metastasis of liver cancer. Downregulation of GBA1 expression in liver cancer cells induces an increase in GlcCer in the plasma membrane, and the reprogramming of GlcCer, which are mainly located in lipid rafts, leads to the enhancement of LRP6. LRP6 is a co-receptor in the Wnt/β-catenin signalling pathway, and increasing the LRP6 level in the plasma membrane induces activation of the Wnt signalling pathway. Reports have shown that activating receptors, including phosphorylation of LRP6, leads to the recruitment of the DVL protein to the plasma membrane, induction of multimerization of the receptor complex and recruitment of components of the β-catenin destruction complex [49]. Subsequently, the proteolysis of β-catenin is inhibited, and non-phospho-β-catenin accumulates in the cytoplasm and is then translocated into the nucleus, as shown in this study. Then, the expression of EMT-associated genes and Wnt target genes is upregulated and the metastasis of liver cancer cells is promoted.

Finally, some results from cell experiments in this study were validated in three human liver cancer tissues. First, we detected the level of GlcCer in three human liver cancer tissues, including one sample with metastatic lesions, by mass spectrometry. As expected, the level of GlcCer was higher in liver cancer tissues than in the paired adjacent nontumorous liver tissues (Fig. 7A). Interestingly, the level of GlcCer in tumour tissue with metastatic lesions appeared to be higher than that in other tumour tissues with nonmetastatic lesions (Fig. 7B). The expression of GBA1 in three cases was also detected by IHC. The results showed that GBA1 was downregulated in liver cancer tissues compared to paired adjacent nontumorous liver tissues (Fig. 7C). Furthermore, we found that the expression of GBA1 in the case with metastatic lesions was lower than that in the other cases without metastatic lesions. We also detected the expression of Wnt signalling pathway proteins and EMT markers by Western blotting, and the results showed that the expression of p-LRP6, c-myc, vimentin, snail and slug was higher in the case with metastatic lesions than in the other cases without metastatic lesions, and E-cadherin appeared opposite change (Fig. 7D). These results indicate that the in vitro and in vivo findings greatly reflect the situation in human liver cancer tissues.

## Discussion

Herein, we demonstrate that GBA1-mediated GlcCer metabolism reprogramming in the plasma membrane promotes liver cancer metastasis via activation of the Wnt/β-catenin signalling pathway. A previous study revealed that GCS was upregulated accompanied by a high level of GlcCer in the liver cancer of the mice lacking *Tsc1* and *Pten* specifically in the liver, and the ratio of GlcCer/Cer was elevated [21]. The study suggests that GCS was upregulated in liver cancer and similar results were observed in breast cancer and colon cancer [39, 40]. Moreover, overexpression of GCS can mediate the balance of GlcCer/Cer and then affects tumorigenesis and the proliferation of liver cancer cells, as we know, GlcCer has the opposite effect on the growth of the cells compared to Cer which induces cell apoptosis and inhibits the growth of cells. Several studies have also shown that GCS-induced GlcCer and/or other GSL metabolic reprogramming is involved in tumour chemotherapy resistance [34, 41]. These studies indicated that the metabolism of GlcCer and other GSLs plays a role in the progression of cancer. However, previous studies have been mainly focused on the effect of GCS on tumours. In this study, we found that the catabolic enzyme GBA1 regulates GlcCer metabolism in the plasma membrane, down regulation of GBA 1 promoting metastasis of liver cancer both in vitro and in vivo. This finding indicates a close relationship between GlcCer metabolism and liver cancer metastasis and suggests that GlcCer can regulate liver cancer metastasis. Importantly, we found that some subspecies of GlcCer, including GlcCer d18:1/14:0, GlcCer d18:1/16:0, and GlcCer d18:1/17:0, were increased in the plasma membrane but appeared to be unchanged in the whole-cell after knockdown of GBA1, and other subspecies of GlcCer showed same trend: The levels of these subspecies were changed in the plasma membrane and whole cell after inhibition of GBA1. These findings indicate that inhibition of GBA1 mainly results in GlcCer metabolism reprogramming in the plasma membrane. Furthermore, when GBA1 was downregulated, GlcCer d18:1/18:0 was the most notably changed tested species in the plasma membrane, suggesting that GlcCer d18:1/18:0 may be the major functional lipid that promotes GBA1-dependent liver cancer metastasis. Performing mechanistic studies, we found that GBA1-dependent GlcCer metabolism reprogramming in the plasma membrane changes the location of LRP6 in the plasma membrane, and leads to an interaction between endogenous GlcCer and LRP6, then promoting the phosphorylation of LRP6 at Ser1490, finally activating the Wnt/β-catenin signalling pathway. In contrast to sphingomyelin, which is distributed approximately equally over the membrane, GSLs, including GlcCer, are primarily located in lipid rafts. One kind of lipid raft called a cholesterol- and sphingolipid-enriched domain is enriched with cholesterol and GSLs, and it serves as receptor signalling platform [32]. Previous studies have shown that cholesterol in the plasma membrane regulates the canonical Wnt signalling pathway [29, 42]. However, the roles of GlcCer, in regulating this signalling pathway remain poorly understood. In this study, we found that the levels of LRP6 in the plasma membrane were increased when GBA1 was knocked down or downregulated when GBA1 was overexpressed. At the same time, metabolic changes were observed in GlcCer, and the Wnt signalling pathway was activated. As GlcCer is mainly located in lipid rafts in the membrane, a change in GlcCer levels leads to a change in the composition of lipid rafts, which results in a change in the location of signalling receptors in the plasma membrane, then leads to an interaction of GlcCer and LRP6. Notably, to our knowledge, this is the first time to be found that GlcCer interacted with protein. Although a change in LRP6 level/location in the plasma membrane after regulation of GBA1 in liver cancer was observed in this study, the mechanism, such as inhibition of receptor internalisation, needs to be studied in greater depth.

As shown in this study, GlcCer is involved in liver cancer metastasis. Previous studies have revealed that lipid metabolism supports the multi-step metastasis cascade [13, 43]. Several studies have demonstrated that GSLs play roles in the metastasis of different cancers [44], but the effect of GlcCer on the metastasis of liver cancer remains unclear. The results of this study indicate that a GBA1-dependent increase in GlcCer can act as a hallmark of liver cancer metastasis.

We also demonstrated that the GBA1 protein is downregulated in liver cancer, especially in patients with vascular invasion. The ROC curve for liver cancer diagnosis was 86% in this study, indicating that GBA1 is a potential diagnostic biomarker for liver cancer. To date, although vascular invasion is a critical risk factor for liver cancer recurrence and poor survival [45, 46], no biomarker of vascular invasion of liver cancer cells has been identified, and the mechanism remains unclear. As shown in this study, GBA1 may be a biomarker of vascular invasion. Furthermore, as GBA1 is expressed at low levels in liver cancer, upregulation of GBA1 may be a potential therapeutic strategy to combat the metastasis of liver cancer. Enzyme replacement therapy is an effective treatment for Gaucher’s disease; hence, whether the drugs used for enzyme replacement therapy in Gaucher’s disease would also be effective treatments for liver cancer metastasis needs to be further studied in basic and clinical research.

As GlcCer has been proved to have a relationship with chemotherapy resistance in cancers [47], we also investigated the relationship between GBA1-dependent GlcCer reprogramming in the liver cancer cell and sorafenib resistance. We chose the sorafenib resistant Huh7 cells (Huh7s) to study the chemoresistance effect of GBA1 in Huh7s. First, we found Huh7s obtained a spindle shape and loose cell-cell contact (Fig. S5A), and the IC50 value (15.527 ± 0.535) showed a shift to a higher concentration compared to its parental line (5.6 ± 0.47) (Fig. S5B). Silence of GBA1 could increase the cell vitality by 4.3-fold in Huh7s under sorafenib exposure, and overexpression of GBA1 revealed opposite effect (Fig. S5C–E), indicating that low expression of GBA1 could increase chemotherapy resistance of liver cancer cells against sorafenib. Besides, we also found downregulation of GBA1 inhibited sorafenib sensitivity of liver cancer cells. As the results showed, when GBA1 was knockdown, IC50 value in HepG2 under sorafenib exposure was obviously increased by 2.5-fold (Fig. S5F); when GBA1 was overexpressed, IC50 value in MHCC-97H under sorafenib exposure was decreased by 3.4-fold (Fig. S5G). All these data indicated that downregulation of GBA1 in liver cancer cell may favour the resistance to chemotherapy, and it is of great significance in the chemotherapy strategy for liver cancer.

In conclusion, we demonstrated that GBA1 is downregulated in liver cancer, and the low expression of GBA1 is associated with vascular invasion and an advanced stage of liver cancer. GBA1-dependent GlcCer metabolism reprogramming in the plasma membrane promotes liver cancer metastasis by activating Wnt/β-catenin signalling. Further mechanistic studies revealed that GlcCer metabolism reprogramming in the plasma membrane results in a change in the location of LRP6 in the plasma membrane, then leads to an interaction of GlcCer and LRP6, which activate the Wnt signalling pathway and then promote the EMT. Upregulation of GBA1 may be a novel therapeutic strategy for combating liver cancer metastasis.

## Material and methods

### Lipidomic analysis

Lipids were extracted from approximately 1 × 10^6^ cells using a modified version of Bligh and Dyer’s method as described previously [48]. Cells were incubated in chloroform:methanol 1:2 (v:v) with 10% deionized water, and then, deionized water and chloroform were added. The samples were then centrifuged, and the lower organic phase containing lipids was extracted.

For lipidomic analyses, polar lipids were analysed using an Exion UPLC system coupled with a triple quadrupole/ion trap mass spectrometer (6500 Plus Qtrap; SCIEX) as described previously [48]. Separation of individual with polar lipids into lipid classes was accomplished with normal phase (NP)-HPLC using a Phenomenex Luna 3-µm silica column (internal diameter of 150 × 2.0 mm). MRM transitions were established for a comparative analysis of various polar lipids. Individual lipid species were quantified by referencing peaks to spiked internal standards.

### Proximity ligation assay (PLA)

Interaction between endogenous GlcCer and LRP6 in situ is detected by PLA in HepG2 liver cancer cells, the protocol is according to the manufacturer’s instruction and previous report with slight modification [38]. Details of PLA are provided in the Supplementary materials and methods.

### Statistics

All data were analysed with GraphPad Prism Version 8.0 software or SPSS 20.0 software. Depending on the data, Student’s *t* test, ROC curve, one-way ANOVA, LSD, chi-square test, or Fisher’s exact probability test was used for comparing differences, and *p* < 0.05 was considered to be significant. Statistical tests and *p* values of each experiment are shown in the legends of the figures.

Further details of materials and methods are provided in the Supplementary materials and methods.

## Supplementary information

Figure S1

Figure S2

Figure S3

Figure S4

Figure S5

Supplemental Figure legends

Supplementary materials and methods

Original Data File

aj-checklist

## Data Availability

All data are available in the main text or supplementary materials.
