# Supplementary figures and images for: GBA1-dependent membrane glucosylceramide reprogramming promotes liver cancer metastasis via activation of the Wnt/β-catenin signalling pathway
Source: Cell Death Dis. 2022 May 30;13(5):508. doi: 10.1038/s41419-022-04968-6 (PMC9151913; doi:10.1038/s41419-022-04968-6)

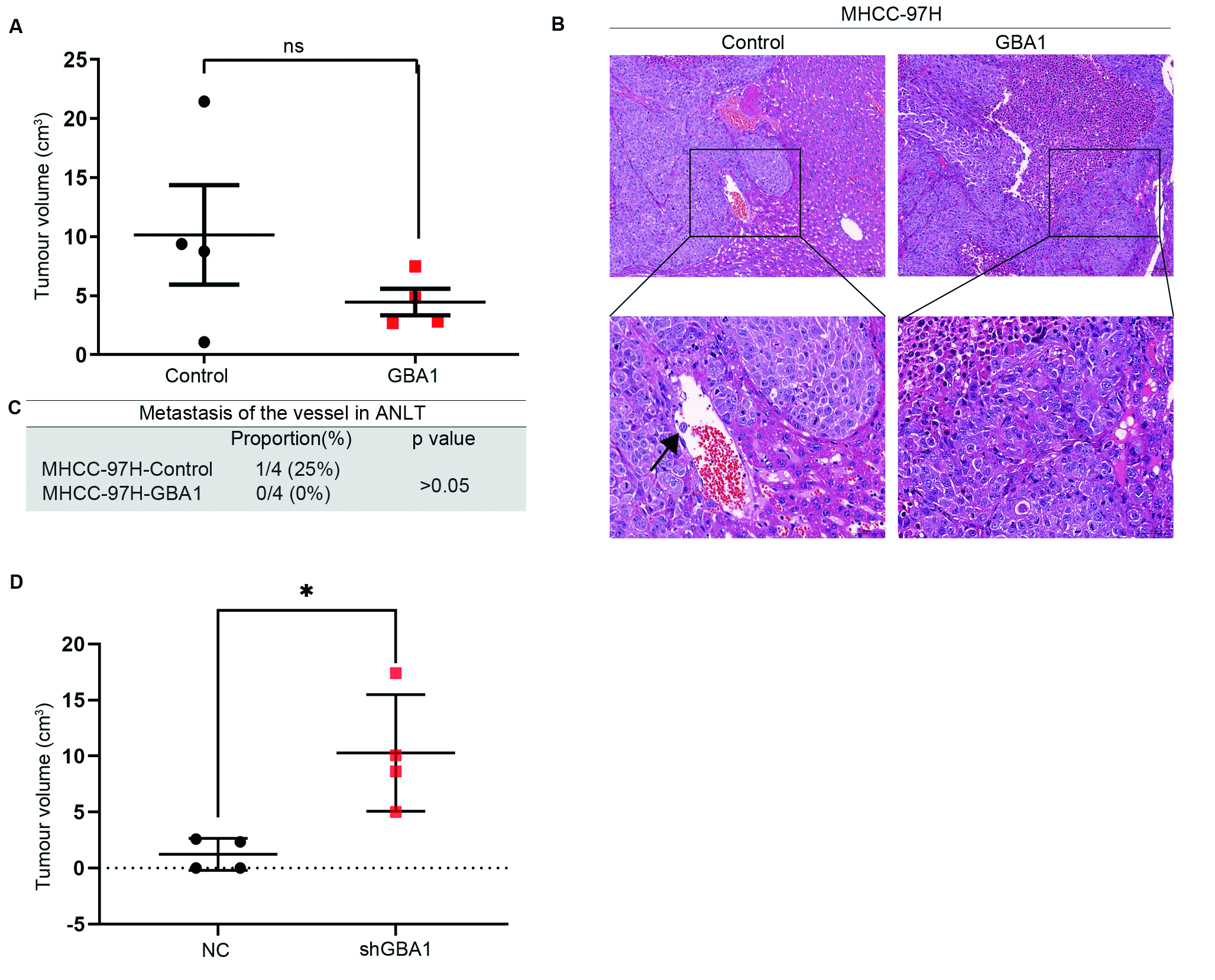

Supplement: Supplementary file 1 — Figure S1 [file 41419_2022_4968_MOESM1_ESM.tif]

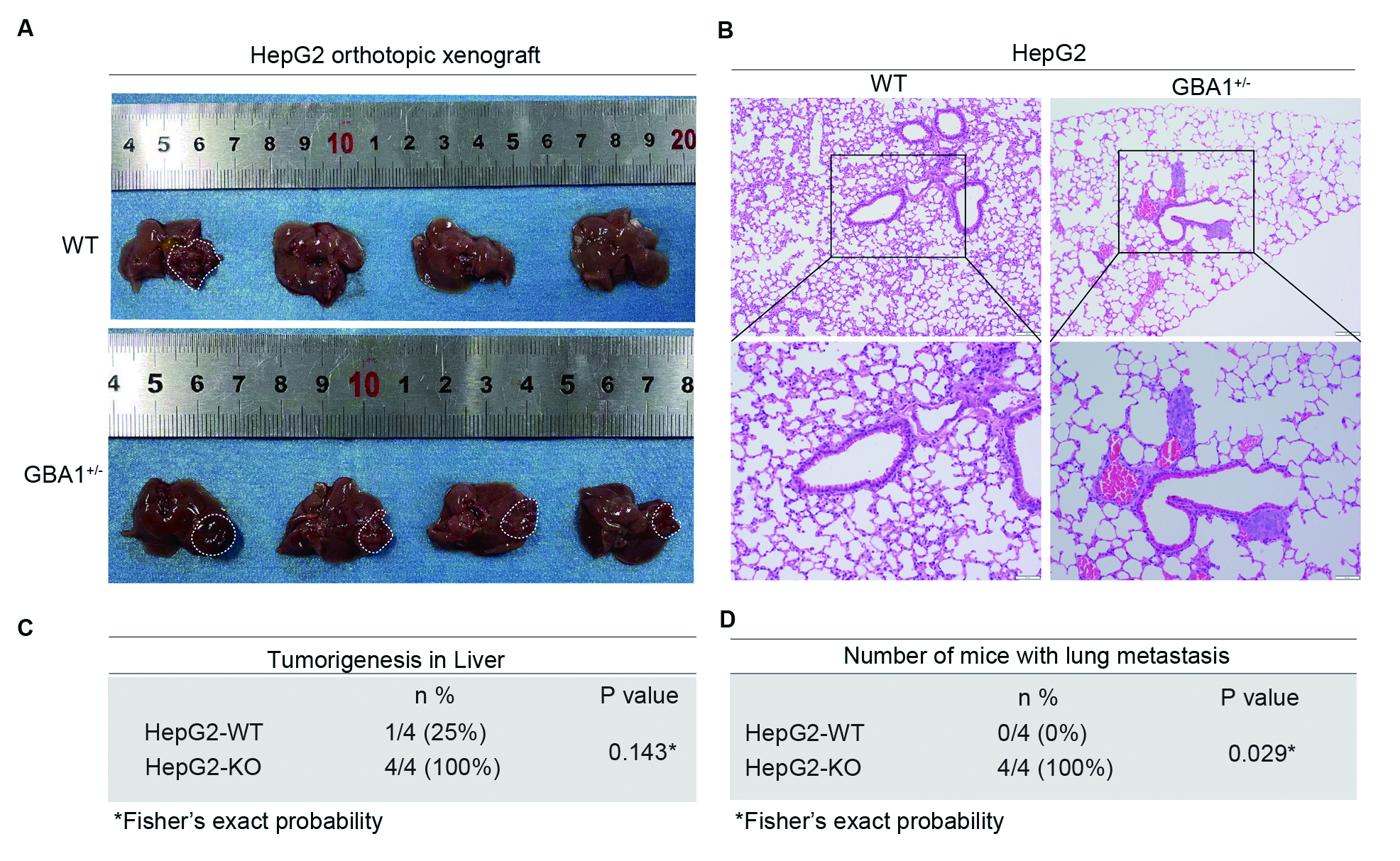

Supplement: Supplementary file 2 — Figure S2 [file 41419_2022_4968_MOESM2_ESM.tif]

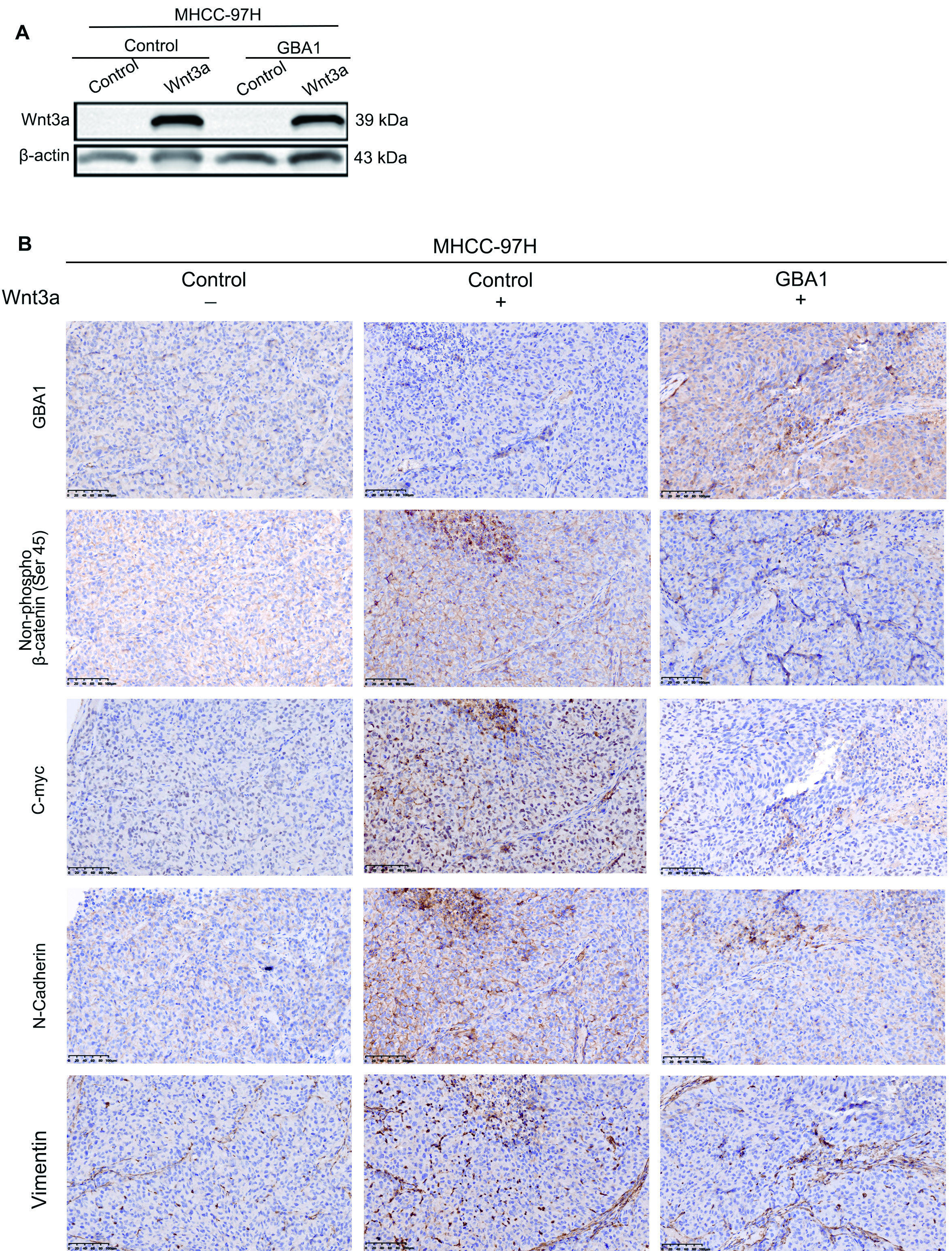

Supplement: Supplementary file 3 — Figure S3 [file 41419_2022_4968_MOESM3_ESM.tif]

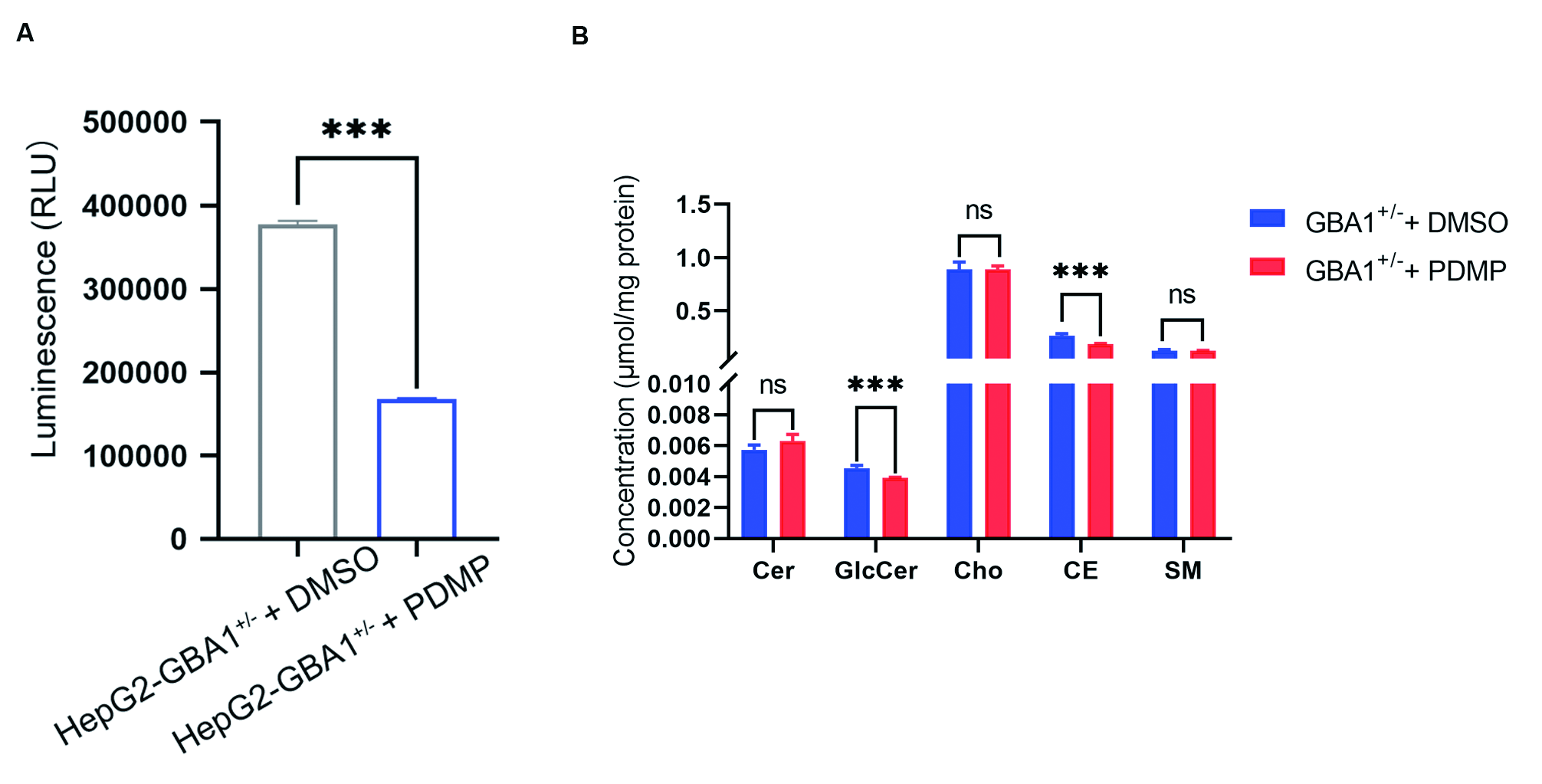

Supplement: Supplementary file 4 — Figure S4 [file 41419_2022_4968_MOESM4_ESM.tif]

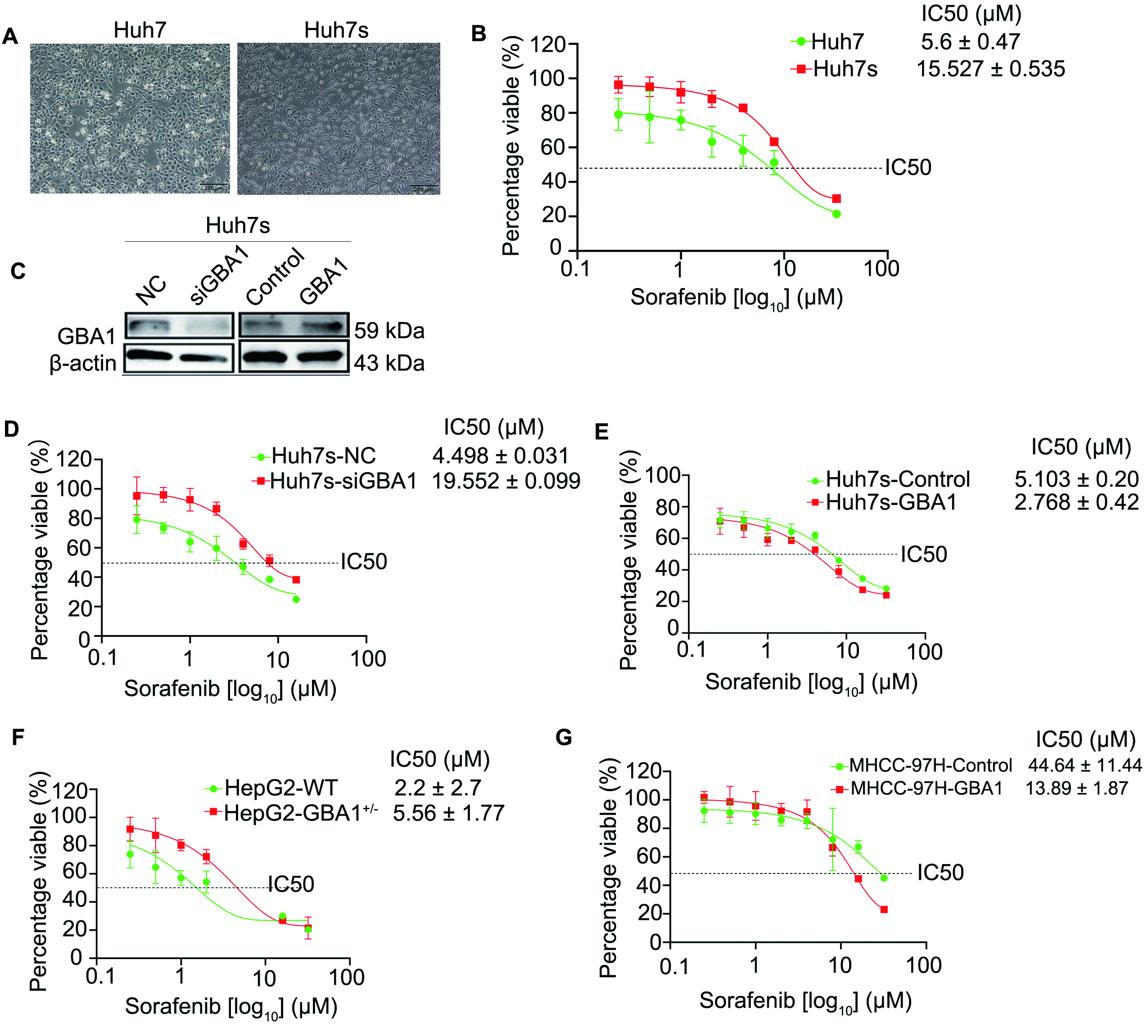

Supplement: Supplementary file 5 — Figure S5 [file 41419_2022_4968_MOESM5_ESM.tif]
