# Supplementary material for: GBA1-dependent membrane glucosylceramide reprogramming promotes liver cancer metastasis via activation of the Wnt/β-catenin signalling pathway
Source: Cell Death Dis. 2022 May 30;13(5):508. doi: 10.1038/s41419-022-04968-6 (PMC9151913; doi:10.1038/s41419-022-04968-6)
Supplement: Supplementary file 6 — Supplemental Figure legends [file 41419_2022_4968_MOESM6_ESM.docx]

**Supplemental figure legends**

**Figure S1 The effects of GBA1 gain- or loss-of-function on the tumour growth and metastasis in vessels in vivo**

(A) To observe the effect of GBA1 gain-of-function on the tumour volume in the orthotopic xenograft model by injecting MHCC-97H cells with lentivirus mediated-GBA1-overexpression. The data are shown as the means ± SD (n=4). Significance was determined by Student’s t-test.

(B-C) To observe the effect of GBA1 gain-of-function using MHCC-97H cells with lentivirus-mediated-GBA1-overexpression on the metastasis in a vessel located at the junction between liver tumour tissue and normal liver tissue. Representative images of vessel metastatic lesions stained by H&E are shown (B), and the proportion of vessel metastatic lesions in the four mice in each group is shown (C). The arrow indicates the metastatic vessel lesions. Significance was determined by Fisher’s exact probability test.

(D) To observe the effect of GBA1 loss-of-function on the tumour volume in the orthotopic xenograft model by injecting Huh7 cells with shRNA-mediated-GBA1-depletion. The data are shown as the means ± SD (n=4). Significance was determined by Student’s t-test.

*, P<0.05; ns, not significant. Scale bars of images in B, 100 μm (upper) and 50 μm (lower).

**Figure S2 GBA1 inhibition promotes the metastasis of liver cancer in vivo**

(A and C) To observe the effect of GBA1 loss-of-function on liver cancer formation in the orthotopic xenograft model using HepG2-WT and HepG2-KO (GBA1^+/-^) cells (A). The data are shown as the percentage of liver tissue with cancer formation (C, n=4). Significance was determined by Fisher’s exact probability test.

(B and D) To observe the effect of GBA1 loss-of-function on spontaneous lung metastasis in the orthotopic xenograft model using HepG2-WT and HepG2-KO (GBA1^+/-^) cells. Representative images of metastatic lung lesions stained by H&E staining are shown (B). The data are shown as the percentage of mice with lung metastasis. Significance was determined by Fisher’s exact probability test.

**Figure S3 Upregulation of GBA1 reduces Wnt/β-catenin signalling pathway and EMT activated by Wnt3a in the orthotopic xenograft model**

A. Through Western blotting to confirm Wnt3a overexpression in the MHCC-97H-Control and MHCC-97H-GBA1 stable cell lines transfected with pLenti-C-mGFP vector with full-length Wnt3a.

B. Through IHC staining to observe upregulation of GBA1 reducing Wnt/β-catenin signalling and EMT activated by Wnt3a in the orthotopic xenograft model. MHCC-97H-Control and MHCC-97H-GBA1 stable cell lines were transfected with pLenti-C-mGFP vector with full-length Wnt3a for 48h, and then these cells (4×10^6^) were injected into the left hepatic lobe of mice in each group to establish orthotopic xenograft model, these mice were sacrificed after 4 weeks, the livers containing tumours were fixed with 4% paraformaldehyde and embedded in paraffin for further IHC staining. Scale bar, 100 μm.

**Figure S4 PDMP inhibits GCS activity and changes the profile of sphingolipid composition in HepG2 cells with GBA1 knockdown**

A. HepG2 cells with GBA1 knockdown were treated by PDMP for 48h and GCS activity was assayed.***P<0.001.

B. HepG2 cells with GBA1 knockdown were treated by PDMP for 48h and sphingolipid was measured by lipidomic analysis. ***P<0.001.

**Figure S5 Low GBA1 inhibits sensitivity of sorafenib in HCC cells**

(A) Morphological comparison between Huh7 (left) and Huh7s (right).

(B) Dose response curve and IC50 determination for sorafenib in Huh7 and Huh7s. Each curve represents the average of values from three independent experiments. Cell proliferation was measured by CCK8 assay.

(C) GBA1 protein levels in Huh7s with GBA1 knockdown by siRNA or GBA1 transient overexpression were determined by Western blotting.

(D-E) Dose response curve and IC50 determination for sorafenib in Huh7s with GBA1 silence (D) or GBA1 overexpression (E). 0.5 × 10^6^ Huh7s cells were plated onto 6-well plates, after 48 hours of transfection with siRNA or plasmid of GBA1, then 3 × 10^3^ cells were plated onto 96-well plates and treated for 48 hours with different sorafenib concentrations ranging from 0 to 32 μM. Each curve represents the average of values from three independent experiments. Cell proliferation was measured by CCK8 assay.

(F-G) Dose response curve and IC50 determination for sorafenib in HepG2 with GBA1-knockdown (F) and MHCC-97H cells with GBA1 stably overexpressed (G). Each curve represents the average of values from three independent experiments. Cell proliferation was measured by CCK8 assay.
