# Supplementary material for: GBA1-dependent membrane glucosylceramide reprogramming promotes liver cancer metastasis via activation of the Wnt/β-catenin signalling pathway
Source: Cell Death Dis. 2022 May 30;13(5):508. doi: 10.1038/s41419-022-04968-6 (PMC9151913; doi:10.1038/s41419-022-04968-6)
Supplement: Supplementary file 7 — Supplementary materials and methods [file 41419_2022_4968_MOESM7_ESM.docx]

**Supplementary materials and methods**

**Cell Lines**

Six liver cancer cell lines (HepG2, Huh7, MHCC-97L, MHCC-97H, SK-Hep1, and the sorafenib resistant Huh7 cells) with different metastatic potentials were used in this study. MHCC-97L and MHCC-97H cells with stepwise increasing metastatic potential were established at Zhongshan Hospital of Fudan University(1-3). The SK-Hep1 cell line was obtained from American Type Culture Collection (Rockville, USA), which was established from ascites of metastatic liver cancer. HepG2 and Huh7 cells were obtained from Shanghai Cell Bank of Chinese Academy of Science (Shanghai, China), the sorafenib resistant Huh7 cells (named Huh7s) were obtained from Bluef (Shanghai) Biotechnology Development Co, Ltd. All cells were authenticated by short tandem repeat (STR) profiling and used within 6 months. HepG2 cells were cultured in EMEM (ATCC) supplemented with 10% FBS (Gibco) and 1% penicillin/streptomycin at 37°C in 5% CO_2_, and other cell lines were maintained in DMEM (Gibco) supplemented with 10% FBS (Gibco) and 1% penicillin/streptomycin at 37°C in 5% CO_2_.

**Animals**

All animal experiments were performed in 5-week-old male nude mice (BALB/c-nu) obtained from Hunan SJA Laboratory Animal Co., Ltd. (Hunan, China) and maintained on a 12 h light-dark cycle at 22°C. Mice were randomly divided into each groups during each animal experiment. All mouse experiments were performed according to protocols approved by the Animal Care and Welfare Committee of Guilin Medical University, and conformed to the guidelines of National Institutes of Health on the ethical use of animals.

**Clinical Specimens**

Tissue samples of liver cancer with portal vein tumour thrombus (PVTT, n=33), liver cancer without portal vein tumour thrombus (non-PVTT, n=33), and paired adjacent nontumorous liver tissues were obtained from the Affiliated Hospital of Guilin Medical University (Guilin, China). Liver cancer with PVTT or non-PVTT was judged by imaging features and pathologic diagnosis after surgical resection. Clinical samples were collected from patients after obtaining informed consent in accordance with a protocol approved by the Ethics Committee of the Affiliated Hospital of Guilin Medical University (Guilin, China).

Another cohort of samples consisted of 3 pairs of liver cancer tissues and paired adjacent nontumorous liver tissues. One case with metastatic lesions (defined as MH, according to imaging features and the pathologic diagnosis after surgical resection) and two cases without metastatic lesions (defined as NMH, according to imaging features and the pathologic diagnosis after surgical resection) were also obtained from the Affiliated Hospital of Guilin Medical University (Guilin, China). Clinical samples were collected from patients after obtaining informed consent in accordance with a protocol approved by the Ethics Committee of the Affiliated Hospital of Guilin Medical University (Guilin, China). The third cohort of independent samples containing 90 liver cancer cases, including liver cancer tissues and paired adjacent nontumorous liver tissues for tissue microarray, was obtained from Shanghai Outdo Biotech (Shanghai, China). The use of these samples for tissue microarray was approved by the Ethics Committee of the Affiliated Hospital of Guilin Medical University (Guilin, China). The fourth cohort of independent samples containing 143 liver cancer cases, including liver cancer tissues and paired adjacent nontumorous liver tissues for tissue microarray, was obtained from Fanpu Biotech, Inc (Guilin, China). The use of these samples for tissue microarray was approved by the Ethics Committee of the Affiliated Hospital of Guilin Medical University (Guilin, China).

**Quantitative RT-PCR**

Total RNA in tissue was extracted by TRIzol, and the total RNA of cells was extracted by FastPure Cell/Tissue Total RNA Isolation Kit V2 (Vazyme, Nanjing, China). Then, the RNA was reverse-transcribed to cDNA. Quantitative RT-PCR was performed according to the manufacturer’s instructions on a Bio-Rad CFX Connect Real-Time System (Bio-Rad, Hercules, CA, USA) using TB Green® Premix Ex Taq™ II (TaKaRa, Dalian, China). The relative expression of the target genes was normalized to that of the control, GAPDH, and the 2^-△△Ct^ method was used to calculate the relative expression of the targets compared to the control.

**Western Blotting**

Total protein was extracted from cells or tissues by RIPA buffer containing protease inhibitor and phosphatase inhibitor cocktail I, and cytoplasmic protein, nuclear protein, and PM protein were extracted from cells following the instructions of a cytoplasmic and nuclear fractionation kit and PM protein isolation and cell fractionation kit. Proteins were separated by electrophoresis on 8% or 10% sodium dodecyl sulfate polyacrylamide gels (SDS-PAGE), transferred onto polyvinylidene fluoride (PVDF) membranes, blocked with 5% non-fat milk in TBST and incubated overnight with primary antibodies at 4°C.

**Immunohistochemistry (IHC)**

IHC staining was performed according to the protocol used in our previous study (4). In brief, after routine dewaxing and hydration, antigen retrieval was performed with 50× EDTA, after which the slides were incubated in 3% H_2_O_2_ solution for 30 min to block endogenous peroxidase activity. Then, the slides were incubated overnight in a humidified chamber with primary antibody in an appropriate concentration at 4°C. The next day, the tissue slides were incubated with biotinylated goat anti-rabbit antibody for 1 h and stained with 3,3´-diaminobenzidine tetrahydrochloride (DAB). Then, the slides were counterstained with haematoxylin. Finally, the stained tissues were analyzed by microscopy, the staining intensity score was determined on a scale of 0 to 3, and the total score of each slide was computed as the sum of the percentage of immunoreactive tumour cells and the staining intensity.

**Cell treatments**

For Wnt 3a supplementation assays, cells were seeded in 6-well plates and then treated with Wnt 3a (100 ng/mL) for 48 h. For PDMP treatment, cells were treated with PDMP (5 μM) for 48 h. For miglustat treatment, cells were treated with miglustat (250 μM) for 48 h.

**Immunofluorescence**

After small-interfering RNA oligonucleotides targeting GBA1 (GenePharma, Jiangsu, China) were transfected into HepG2 cells using the Lipofectamine 3000 reagent (Invitrogen) according to the manufacturer’s protocol for 48 h, suitable quantity of cells was planted into 24 well plates for 24 h. Then the cells were fixed by 4% PFA for 10 min, followed by permeabilization using 0.1% Triton X-100 for 15 min and finally stained using LRP6 antibody followed by appropriated secondary antibodies and DAPI. Images were observed and analyzed using confocal microscopy. The following sequences were used in this experiment: GBA1 siRNA sense, GCUACUCCAUUCACACCUATT; anti-sense, UAGGUGUGAAUGGAGUAGCTT.

**Animal studies**

The mice were divided randomly into different groups, and orthotopic xenograft models were established by injecting different liver cancer cells (4×10^6^) into the left hepatic lobe of mice in each group (n=4), and these mice were sacrificed after 4-6 weeks. The livers containing tumours and lungs were removed, the proportion of nontumorous liver tissue was calculated, and the tumour volume was calculated using the formula: volume = (Length ×Width^2^)/2. Then, the livers containing tumours and lungs were fixed with 4% paraformaldehyde and embedded in paraffin. Consecutive sections in every lung tissue block were prepared and stained with haematoxylin and eosin, and sections were prepared from liver tumour-containing blocks and stained with haematoxylin and eosin. The number of lung metastatic lesions and the number of metastatic lesions in the vasculature of adjacent nontumorous liver tissues were calculated.

**Gene expression and RNA interference**

The lentivirus GBA1 expression plasmid LV-EFS>hGBA-CMV>EGFP/T2A/Puro was generated by cloning the full-length coding sequence of GBA1 into a LV-CMV>EGFP/T2A/Puro vector. shRNA directed against the GBA1 gene was added to a pLV3ltr-ZsGreen-Puro-U6 vector. For overexpression of GBA1, lentiviral plasmids were transfected into HEK293T cells, and the supernatant medium containing virus was then collected. MHCC-97H cells were infected with this collected lentivirus. After MHCC-97H cell infection with lentivirus, puromycin was used to select a stable cell line. To deplete the GBA1 gene, a pLV3ltr-ZsGreen-Puro-U6-GBA1 plasmid was transfected into Huh7 cells using Lipofectamine 3000 (Invitrogen, Carlsbad, CA, USA) according to the manufacturer’s instructions, and puromycin was used to select a stable cell line. For overexpression of Wnt3a, full-length Wnt3a (Myc-DDK tagged, Origene, shanghai, China) was inserted into pLenti-C-mGFP vector (Origene, shanghai, China), and MHCC-97H-control or MHCC-97H-GBA1 stable cell lines was chosen for Wnt3a overexpression using Lipofectamine 3000 reagents (Invitrogen) according to the manufacturer’s instructions.

**CRISPR/Cas9-mediated knockout of gene expression**

The CRISPR/Cas9-gRNA system was used to knock out the GBA1 gene in HepG2 cells. gRNAs were designed as shown in the KEY RESOURCES TABLE — Oligonucleotides. Then, the Cas9 and gRNA were transferred into HepG2 cells by electroporation. After the transfer of Cas9 and gRNA, the monoclonal cells were amplified. A DNA sequence analysis was performed to confirm the knockout of GBA1, and PCR was performed to detect the genotype of the cells.

**In vitro migration and invasion assays**

The migration and invasion experiments were performed with 24-well migration assay inserts and invasion assay inserts (Corning, NY, USA). Different liver cancer cells were seeded in the top chamber in FBS-free DMEM (for MHCC-97H) or EMEM (for HepG2) at a 5×10^4^ cell concentration (MHCC-97H cells) or at an 8×10^4^ cell concentration (HepG2 cells). The bottom chamber was filled with DMEM containing 10% FBS (MHCC-97H) or EMEM containing 10% FBS (HepG2). After 24 h (migration) or 48 h (invasion) of cells incubation at 37 ℃ in a humidified incubator containing 5% CO_2_, the cells were fixed with 4% paraformaldehyde and stained with 0.1% hexamethyl pararosaniline (Solarbio Life Science, China).

**Cell fraction extraction**

Cytoplasmic and nuclear fractionation extraction was performed following the instructions of a Minute™ cytoplasmic and nuclear fractionation kit (Invent Biotechnologies, China). Cells were washed with cold PBS, and then, the appropriate amount of cytoplasmic extraction buffer was added to the cells. Cell lysate was transferred to a prechilled 1.5-ml microcentrifuge tube and centrifuged for 5 min at 16000×g in a microcentrifuge at 4°C. The supernatant contained the cytosolic fraction, and the precipitate contained the nuclear fraction. Appropriate amounts of nuclear extraction buffer were added to the nuclear fraction. After complete lysis of nuclei, the nuclear extract was transferred to a prechilled filter cartridge centrifuge and spun at 16000×g at 4°C in a microcentrifuge for 30 seconds, and the nuclear fraction was poured into a collection tube.

PM isolation was performed following the instructions of the Minute™ plasma membrane protein isolation and cell fractionation kit (Invent Biotechnologies, China). Liver cancer cells (40-50×10^6^) were harvested, resuspended in buffer in the first step (in buffer A), transferred to a filter cartridge, and centrifuged at 16000×g for 30 min. The pellet was resuspended and centrifuged at 700×g, and the supernatant was poured to a 1.5-ml microcentrifuge tube and centrifuged at 16000×g and 4 ℃ for 30 min. Then, in the second step, the total membrane fraction was resuspended (in buffer B) and centrifuged at 7800 ×g and 4 ℃ for 20 min. Then, the supernatant was poured into a 2.0-ml microcentrifuge tube to which 1.6 ml of cold PBS was added and centrifuged at 16000×g for 30 min; the pellet consists of PM.

**Determination of GCS activity**

Isolation of microsomal fractions was performed according to the instructions of microsome isolation kit (ab206995, ABCam, Massachusetts, US). HepG2-GBA1^+/-^ cells (3.8 x 10^7^) treated with 5 μM PDMP for 48 h were placed on ice, washed with ice-cold PBS twice and scraped into 1 mL of ice-cold homogenization buffer. After homogenization in pre-chilled Dounce homogenizer (25 strokes), cell debris was removed by 10,000 x g for 15 minutes centrifugation at 4 °C, then the supernatant was centrifuged at 20,000 x g for 20 minutes at 4 °C. Finally, the pellet was resuspended in 500 μL of Storage Buffer.

GCS activity assay was performed based on the method as previously described (5). The enzyme activity was determined using C6-ceramide and UDP-glucose as substrates following the instructions of UDP-Glo™ Glycosyltransferase Assay kit (Promega, Madison, WI, USA). Briefly, 100 μg of microsomal protein extract, 5 μL C6-ceramide (1mM), 100 x UDP-glucose, and 25 μL UDP detection reagent were used for each assay. After incubation of aforementioned microsome and reagents mixture for 1 h at 37 °C in the dark with gentle shaking, GCS activity was measured by determination of the luminescence in the mixture with a plate-reading luminometer.

**In vitro determination of drug resistance assays**

Sorafenib (MedChemExpress, Monmouth Junction, NJ) was dissolved in DMSO at a final concentration of 10 mM. Cell viability was determined by CCK8 assay. Briefly, to determine the dose response curve and IC50 for sorafenib in Huh7, Huh7s, HepG2-WT, HepG2-GBA1^+/-^, MHCC-97H-control, and MHCC-97H-GBA1 cells, the above cell lines were cultured in 96-well plates at 3 × 10^3^ cells per well, and for dose response curve and IC50 determination for sorafenib in Huh7s with GBA1 knockdown and Huh7s cells overexpressing GBA1, 0.5 × 10^6^ Huh7s were plated onto 6-well plates, after 48 hours of transfection with siRNA or plasmid of GBA1, then 3 × 10^3^ cells were plated onto 96-well plates. After that, the cells were incubated with different sorafenib concentrations ranging from 0 to 32 μM for 48 hours. CCK8 reagent was added at a final concentration of 1% and after 1 h of incubation (37ºC, 5% CO_2_) the absorbance was measured at 450 nm. Prism Software (La Jolla, USA) was used to calculate drug half maximal inhibitory concentration (IC50).

**Proximity ligation assay (PLA)**

PLA procedure mainly includes four steps. The first one is cell preparation. 1 × 10^5^ cells per well were seeded in a 24-well plate overnight, fixed with 4% paraformaldehyde (PFA) for 20 minutes at room temperature (RT), incubated in ice-cold 100% methanol for 30 minutes, and blocked cells with Duolink® block solution for 1 h at RT. The second step is antibody incubation. Dilute the primary antibody against GlcCer (Glycobiotech GmbH, Rabbit) or LRP6 (Invitrogen, mouse) in Duolink® antibody dilution buffer. Cells were incubated with the diluted antibody overnight at 4°C, and incubated PLA probes for 20 min at RT which was prepared according to the instructions as below: 150 μL total volume per reaction including 30 μL PLUS antibody, 30 μL MINUS antibody, and 90 μL Duolink® ab dilution buffer. After that, secondary antibody mixture was incubated at 37°C for 1 h. The third step is ligation and amplification. The cells were incubated ligation mixture per reaction including 30 μL (5×) ligation stock, 116.3 μL distilled water, and 3.7 μL ligase, at 37°C for 30 min, then incubated reaction amplification mixture including 30 μL (5×) amplification stock, 118.1 μL distilled water, and 1.9 μL ligase, for 100 min at 37°C. Then washed slides twice with 1× buffer B each for 10 min, and wash slides once with 0.01× buffer B for 1 min. Then mount the slides using Prolong Gold mounting medium with DAPI for nucleus staining. The last step is image analysis, the fluorescent images were analyzed by confocal microscopy.

**Key resources table**

| REAGENT or RESOURCE | SOURCE | IDENTIFIER |
| --- | --- | --- |
| **Antibody** | | |
| Non-phospho (Active) β-Catenin (Ser45) | Cell Signaling Technology | Cat#19807; RRID: AB_2650576 |
| Cyclin D1 (E3P5S) XP^®^ Rabbit mAb | Cell Signaling Technology | Cat#55506; RRID: AB_2827374 |
| Epithelial-Mesenchymal Transition (EMT) Antibody Sampler Kit | Cell Signaling Technology | Cat#9782; RRID: AB_10828222 |
| Anti-alpha 1 Sodium Potassium ATPase | ABCam | Cat#ab7671; RRID: AB_306023 |
| Rabbit monoclonal [Y69] to c-Myc | ABCam | Cat#ab32072; RRID: AB_731658 |
| Mouse monoclonal [2E2] to GBA | ABCam | Cat#ab55080; RRID: AB_2109076 |
| Rabbit polyclonal to GBA | ABCam | Cat#ab175869; |
| Rabbit monoclonal [EPR22910-39] to LRP6 | ABCam | Cat#ab231779; RRID: AB_231779 |
| Rabbit Anti-GlcCer | Glycobiotech GmbH | RAS_0011; |
| Rabbit monoclonal [EPR8985(B)] to Lamin B1 | ABCam | Cat#ab133741; RRID: AB_2616597 |
| Rabbit monoclonal [EP2360Y] to LRP6 (phospho S1490) | ABCam | Cat#ab76417; RRID: AB_1523898 |
| Anti-Wnt3a [EPR21889] | ABCam | Cat#ab219412 |
| **Biological Samples** | | |
| Human HCC tissue microarrays, containing 90 cases of HCC samples, with 90 matched adjacent normal liver tissues | Shanghai Outdo Biotech Company | HLivH180Su07 |
| Human HCC tissue microarrays, containing 143 cases of HCC samples, with 143 matched adjacent normal liver tissues | Fanpu Biotech, Inc | LVC1504; LVC1505 |
| Human HCC tissue, 46 cases HCC tissues, with matched adjacent normal liver tissues | Guilin Medical University | N/A |
| Human HCC tissues, 1 case metastatic HCC tissue and 2 cases non-metastatic HCC tissues, with matched adjacent normal liver tissues | Guilin Medical University | N/A |
| **Chemicals, Peptides, and Recombinant Proteins** | | |
| D-threo-PDMP, Glycosylceramide synthase inhibitor | ABCam | Cat#ab144052 |
| Recombinant Human Wnt-3a Protein | R&D Systems | Cat#NP_149122 |
| HRP-Conjugated Alpha Tubulin Monoclonal Antibody | Proteintech | Cat#HRP-66031 |
| Mouse Anti-β actin mAb | ZSGB-Bio | TA-09 |
| PMSF (100 mM) | Solarbio life science | P0100 |
| Phosphatase Inhibitor Cocktail I | MedChemExpress | HY-K0021 |
| Lipofectamine 3000 | Thermo Fisher Scientific | L3000015 |
| Puromycin, dihydrochloride | Solarbio life science | P8230 |
| BSA | Sigma-Aldrich | A1933 |
| C6-ceramide | Avanti Polar Lipids | 860506 |
| miglustat | MedChemExpress | HY-17020 |
| **Critical Commercial Assays** | | |
| TB Green® Premix Ex Taq™ II | Takara | RR820Q |
| EndoFree Mini Plasmid Kit II | TIANGEN | DP118 |
| FastQuant RT Kit (With gDNase) | TIANGEN | KR106 |
| Minute™ Plasma Membrane Protein Isolation and Cell Fractionation Kit | Invent biotechnologies | SM-005 |
| Minute™ Cytoplasmic and Nuclear Fractionation kit | Invent biotechnologies | SC-003 |
| Minute™ Denaturing Protein Solubilization Reagent | Invent biotechnologies | WA-009 |
| FastPure® Cell/Tissue Total RNA Isolation Kit V2 | Vazyme | RC112-01 |
| Invasion assay inserts | Corning | 354480 |
| Migration assay inserts | Corning | 3422 |
| Duolink® In Situ PLA ® Probe Anti-Rabbit PLUS | Sigma | DUO92002 |
| UDP-Glo™ Glycosyltransferase Assay | Promega | V6961 |
| UDP detection regent | Promega | V7091 |
| Microsome isolation kit | ABCam | ab206995 |

| **Experimental Models: Cell Lines** | | |
| --- | --- | --- |
| Human: MHCC-97H | Established by Liver Cancer Institute, Fudan University (Shanghai, China) | N/A |
| Human: MHCC-97L | Established by Liver Cancer Institute, Fudan University (Shanghai, China) | N/A |
| Human: Huh7 | ATCC | PTA-4583™ |
| Human: HepG2 | National Collection of Authenticated Cell Cultures | SCSP-510 |
| Human: SK-Hep1 | ATCC | HTB-52™ |
| Human: Huh7s (the sorafenib resistant Huh7 cells) | Bluefcell (Shanghai) Biotechnology Development Co, Ltd | BFN6021426 |
| **Experimental Models: Organisms/Strains** | | |
| Mouse: BALB/c-nu | HUNAN SJA LABORATORY ANIMAL CO., LTD | N/A |
| **Oligonucleotides** |  |  |
| HepG2 gRNA-A1: AATGTGGGAGACCGGGCCATGGG | This paper | N/A |
| HepG2 gRNA-A2: ATTTCACAGGGCATTAAAACAGG | This paper | N/A |
| HepG2 gRNA-B1: CCTCAACTACTCTCCTGGGCAGG | This paper | N/A |
| HepG2 gRNA-B2: AGAAGTCAGGGTCCAAAGAAAGG | This paper | N/A |
| GAPDH qPCR forward primer:  CAGGAGGCATTGCTGATGAT | This paper | N/A |
| GAPDH qPCR reverse primer:  GAAGGCTGGGGCTCATTT | This paper | N/A |
| GBA1 qPCR forward primer:  GGAGCAGCAGTGTCAGCATCAG | This paper | N/A |
| GBA1 qPCR reverse primer:  GCTGGTGCCATAGGAATCATCTGG | This paper | N/A |
| WNT1 qPCR forward primer:  ATCCATCTCTCCCACCTCCTAC | This paper | N/A |
| WNT1 qPCR reverse primer:  GAATCTTTCTCTCACCCTCTGG | This paper | N/A |
| WNT2 qPCR forward primer:  GTGATGTGTGACAATGTGCCA | This paper | N/A |
| WNT2 qPCR reverse primer:  GTTGCAGTTCCAGCGATGC | This paper | N/A |
| WNT2B qPCR forward primer:  TGTGCGGCGGTTTCTGAAGC | This paper | N/A |
| WNT2B qPCR reverse primer:  CCTGTGCGGCGGAAATCTGAG | This paper | N/A |
| WNT3 qPCR forward primer:  AGCGTAGCAGAAGGTGTGAAG | This paper | N/A |
| WNT3 qPCR reverse primer:  CCAGGTGGCCCCTTATGATG | This paper | N/A |
| WNT3A qPCR forward primer:  TCGGAGATGGTGGTAGAGAAAC | This paper | N/A |
| WNT3A qPCR reverse primer:  TCGCAGAAGTTGGGTGAGG | This paper | N/A |
| WNT4 qPCR forward primer:  GCTGGAGAAGTGCGGCTGTG | This paper | N/A |
| WNT4 qPCR reverse primer:  CACCGTAGGCGATGTTGTCAGAG | This paper | N/A |
| WNT5A qPCR forward primer:  GACTTCCGCAAGGTGGGTGATG | This paper | N/A |
| WNT5A qPCR reverse primer:  GTCTTGTGTGGTGGGCGAGTTG | This paper | N/A |
| WNT5B qPCR forward primer:  CTGCTGTTCACGGCTGCTCTG | This paper | N/A |
| WNT5B qPCR reverse primer:  GGGCTGGGCACCGATGATAAAC | This paper | N/A |
| WNT6 qPCR forward primer:  GCCAGTTCCAGTTCCGCTTCC | This paper | N/A |
| WNT6 qPCR reverse primer:  CGTCTCCCGAATGTCCTGTTGC | This paper | N/A |
| WNT7A qPCR forward primer:  GGCTACGTGCTCAAGGACAAGTAC | This paper | N/A |
| WNT7A qPCR reverse primer:  GCGGTACGACAGTGGCTTCTTG | This paper | N/A |
| WNT7B qPCR forward primer:  GCGTGCCATCTGCCAGAGTC | This paper | N/A |
| WNT7B qPCR reverse primer:  CAGTTCCAGCGTCCGAAGCG | This paper | N/A |
| WNT8A qPCR forward primer:  ACAGAACAGCCACAACACATCCAG | This paper | N/A |
| WNT8A qPCR reverse primer:  CACACTTGACCGTACAGCACCAC | This paper | N/A |
| WNT8B qPCR forward primer:  CGCCATCGCCGACACCTTTC | This paper | N/A |
| WNT8B qPCR reverse primer:  CCCAGCAGCCCTAGCGTTTTG | This paper | N/A |
| WNT9A qPCR forward primer:  CACCGTGAGAAGAACTGCGAGAG | This paper | N/A |
| WNT9A qPCR reverse primer:  CACCGTGAGAAGAACTGCGAGAG | This paper | N/A |
| WNT9B qPCR forward primer:  GTGCGGTGACAACCTCAAGTACAG | This paper | N/A |
| WNT9B qPCR reverse primer:  GTGGGTATTGTGGGCGTCTGC | This paper | N/A |
| WNT10A qPCR forward primer:  GCGGCAGATGGAGGTGTGTG | This paper | N/A |
| WNT10A qPCR reverse primer:  TGGTGTTGGCATTCGTGGATGG | This paper | N/A |
| WNT10B qPCR forward primer:  GCTGTGGCTGTGGCTGGAAG | This paper | N/A |
| WNT10B qPCR reverse primer:  CAGGGCTGGGCAGAGAGTGG | This paper | N/A |
| WNT11 qPCR forward primer:  TGCTGACCTCAAGACCCGATACC | This paper | N/A |
| WNT11 qPCR reverse primer:  AGACGAGTTCCGAGTCCTTCACAG | This paper | N/A |
| WNT16 qPCR forward primer:  ATCGGAAACACCACGGGCAAAG | This paper | N/A |
| WNT16 qPCR reverse primer:  CAGCGGCAGTCTACTGACATCAAC | This paper | N/A |
| FZD1 qPCR forward primer:  ATGAAGCACGATGGCACCAAGAC | This paper | N/A |
| FZD1 qPCR reverse primer:  GAAGTAGCAGGCGATGACGATGG | This paper | N/A |
| FZD2 qPCR forward primer:  GACATCGCCTACAACCAGACCATC | This paper | N/A |
| FZD2 qPCR reverse primer:  GCACCTTCACCAGCGGATAGAAC | This paper | N/A |
| FZD3 qPCR forward primer:  TGAAGATCGAGTAGCCTGCAATGC | This paper | N/A |
| FZD3 qPCR reverse primer:  CCACCATACACTGCCAGCCATAG | This paper | N/A |
| FZD4 qPCR forward primer:  CGTGACCAAGATGCCCAACCTG | This paper | N/A |
| FZD4 qPCR reverse primer:  TGGAGCAGCCGTACTGGATGAG | This paper | N/A |
| FZD5 qPCR forward primer:  GCACAACCACATCCACTACGAGAC | This paper | N/A |
| FZD5 qPCR reverse primer:  AGGAACCAGGTGAGCGACAGG | This paper | N/A |
| FZD6 qPCR forward primer:  GCCACTGTGCCTTTGTGTGTTTG | This paper | N/A |
| FZD6 qPCR reverse primer:  AAGCCGCTGAAGACTCCAATTCG | This paper | N/A |
| FZD7 qPCR forward primer:  CGGCATCACCACTGGCTTCTG | This paper | N/A |
| FZD7 qPCR reverse primer:  CCTTGCTGCTGTGGCTAAGTCTG | This paper | N/A |
| FZD8 qPCR forward primer:  TCGCCACCGTCTCCACCTTC | This paper | N/A |
| FZD8 qPCR reverse primer:  AGCCCACCGACACGAAGAGG | This paper | N/A |
| FZD9 qPCR forward primer:  GGCGGCACCAACACAGAGAAG | This paper | N/A |
| FZD9 qPCR reverse primer:  AAGTCCATGTTGAGGCGTTCGTAG | This paper | N/A |
| FZD10 qPCR forward primer:  GCGAGGCAGCCATCCAGTTG | This paper | N/A |
| FZD10 qPCR reverse primer:  CGTACAGCGAGCACAGGAAGAAG | This paper | N/A |
| LRP5 qPCR forward primer:  CTCTACTGGACCGACCTGGACAC | This paper | N/A |
| LRP5 qPCR reverse primer:  GCTGTACTGCGTCAGACCGAAC | This paper | N/A |
| LRP6 qPCR forward primer:  AATCGGCAGGCAGTGGTTAAAGG | This paper | N/A |
| LRP6 qPCR reverse primer:  AATGGATTTCACGCAGACCCTCAC | This paper | N/A |
| **Recombinant DNA** | | |
| LV-EFS>hGBA-CMV>EGFP/T2A/Puro | cyngen |  |
| **Software and Algorithms** | | |
| GraphPad Prism Version 8.0 | GraphPad Prism Software Inc | <http://www.gaphpad.com/scientific>-software/prism |
| SPSS 20.0 | IBM | https://www.ibm.com/cn-zh/products/spss-statistics |

1. Lu M, Zhu WW, Wang X, Tang JJ, Zhang KL, Yu GY, et al. ACOT12-Dependent Alteration of Acetyl-CoA Drives Hepatocellular Carcinoma Metastasis by Epigenetic Induction of Epithelial-Mesenchymal Transition. Cell Metab. 2019;29(4):886-900 e5.

2. Li Y, Tian B, Yang J, Zhao L, Wu X, Ye SL, et al. Stepwise metastatic human hepatocellular carcinoma cell model system with multiple metastatic potentials established through consecutive in vivo selection and studies on metastatic characteristics. J Cancer Res Clin Oncol. 2004;130(8):460-8.

3. Lin L, Ding Y, Wang Y, Wang Z, Yin X, Yan G, et al. Functional lipidomics: Palmitic acid impairs hepatocellular carcinoma development by modulating membrane fluidity and glucose metabolism. Hepatology. 2017;66(2):432-48.

4. Lin M, Liao W, Dong M, Zhu R, Xiao J, Sun T, et al. Exosomal neutral sphingomyelinase 1 suppresses hepatocellular carcinoma via decreasing the ratio of sphingomyelin/ceramide. FEBS J. 2018;285(20):3835-48.

5. Bourteele S, Hausser A, Doppler H, Horn-Muller J, Ropke C, Schwarzmann G, et al. Tumor necrosis factor induces ceramide oscillations and negatively controls sphingolipid synthases by caspases in apoptotic Kym-1 cells. J Biol Chem. 1998;273(47):31245-51.
