# Supplementary material for: GBA1-dependent membrane glucosylceramide reprogramming promotes liver cancer metastasis via activation of the Wnt/β-catenin signalling pathway
Source: Cell Death Dis. 2022 May 30;13(5):508. doi: 10.1038/s41419-022-04968-6 (PMC9151913; doi:10.1038/s41419-022-04968-6)
Supplement: Supplementary file 8 — Original Data File [file 41419_2022_4968_MOESM8_ESM.pdf]

Figure 1 Low expression of GBA1 is associated with the metastatic potential of liver cancer cells

(I) Western blot analysis of GBA1 protein in liver cancer cell lines with different metastatic potentials

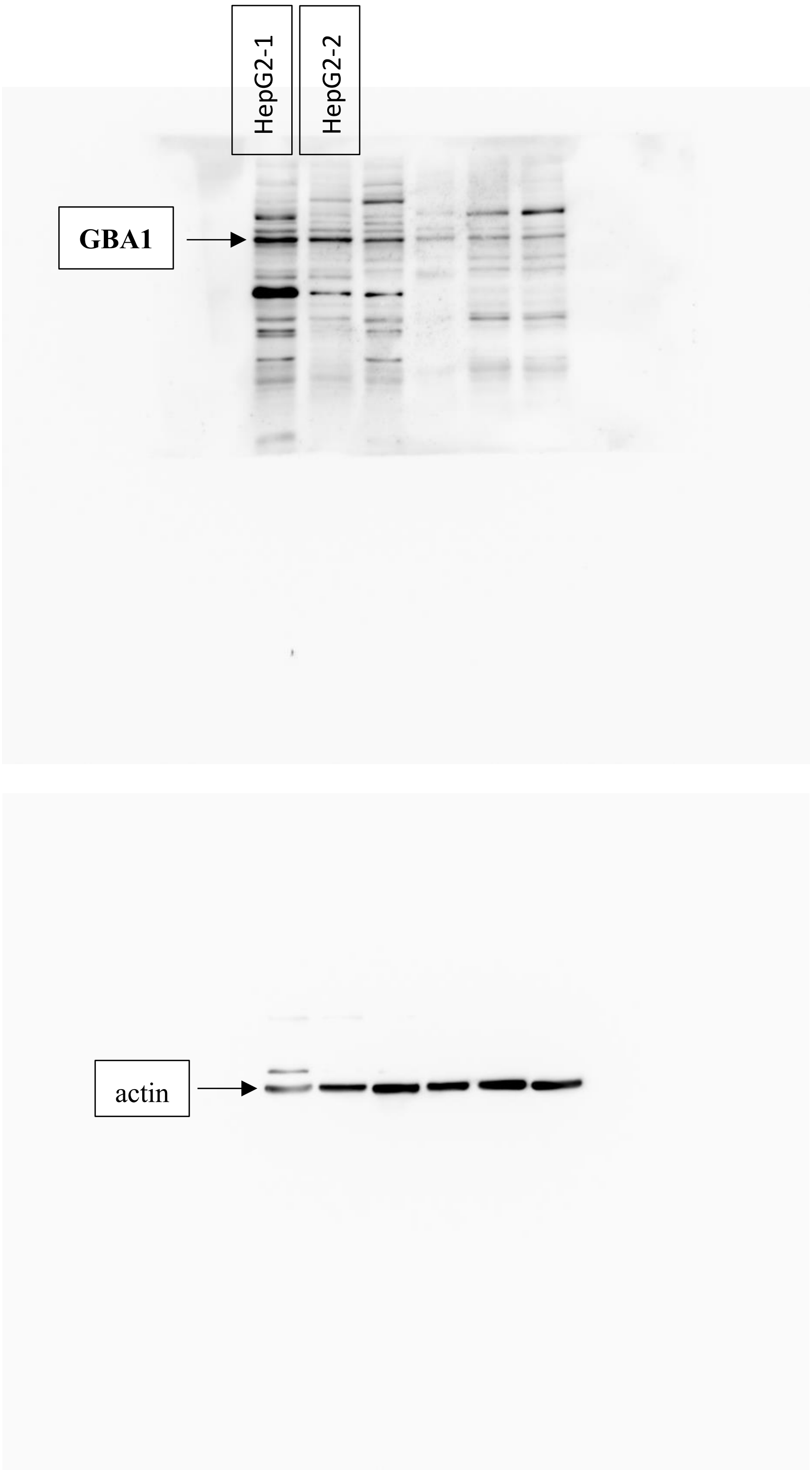

Figure 2 Downregulation of GBA1 promotes metastasis of liver cancer in vitro and in vivo

(A) Confirmation of GBA1 overexpression in MHCC-97H cells.

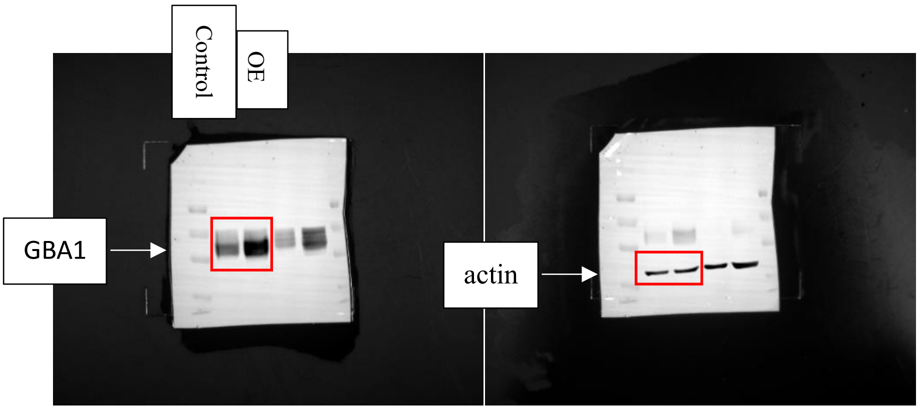

(B)Confirmation of GBA1 knockdown in HepG2 liver cancer cell lines.

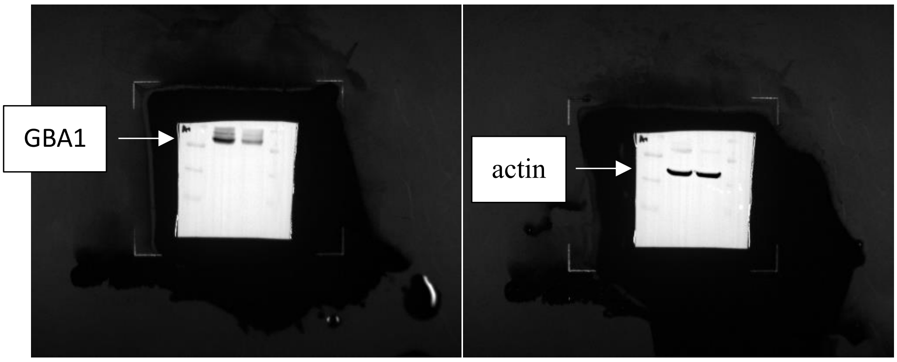

Figure 3 Low expression of GBA1 promotes the epithelial-mesenchymal transition (EMT) via activation of the Wnt/β-catenin signalling pathway

(A) The expression of EMT markers in MHCC-97H cells with stable GBA1 overexpression as detected by Western blotting.

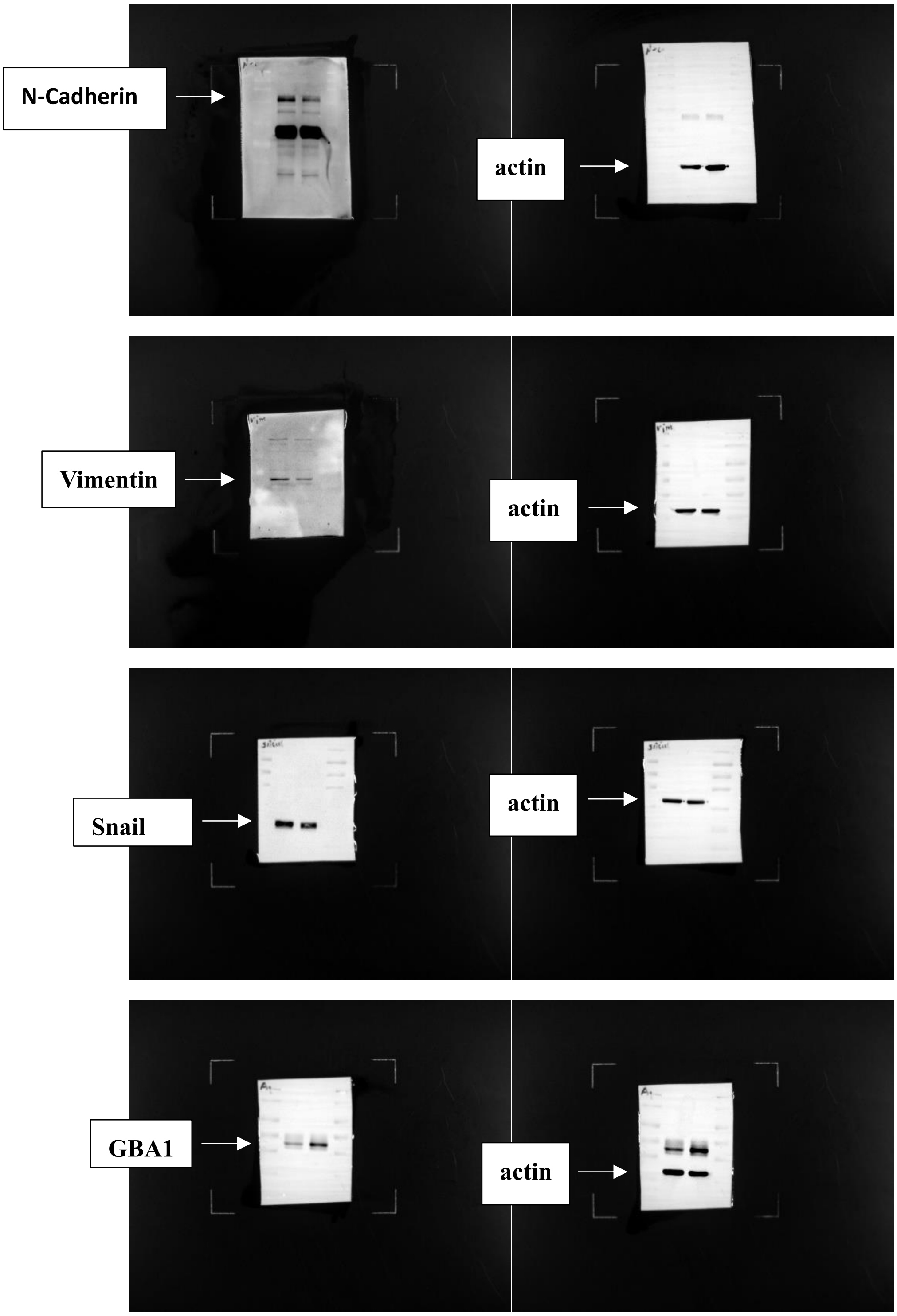

(D) The expression of EMT markers in HepG2 cells with stable GBA1 downregulation as detected by Western blotting.

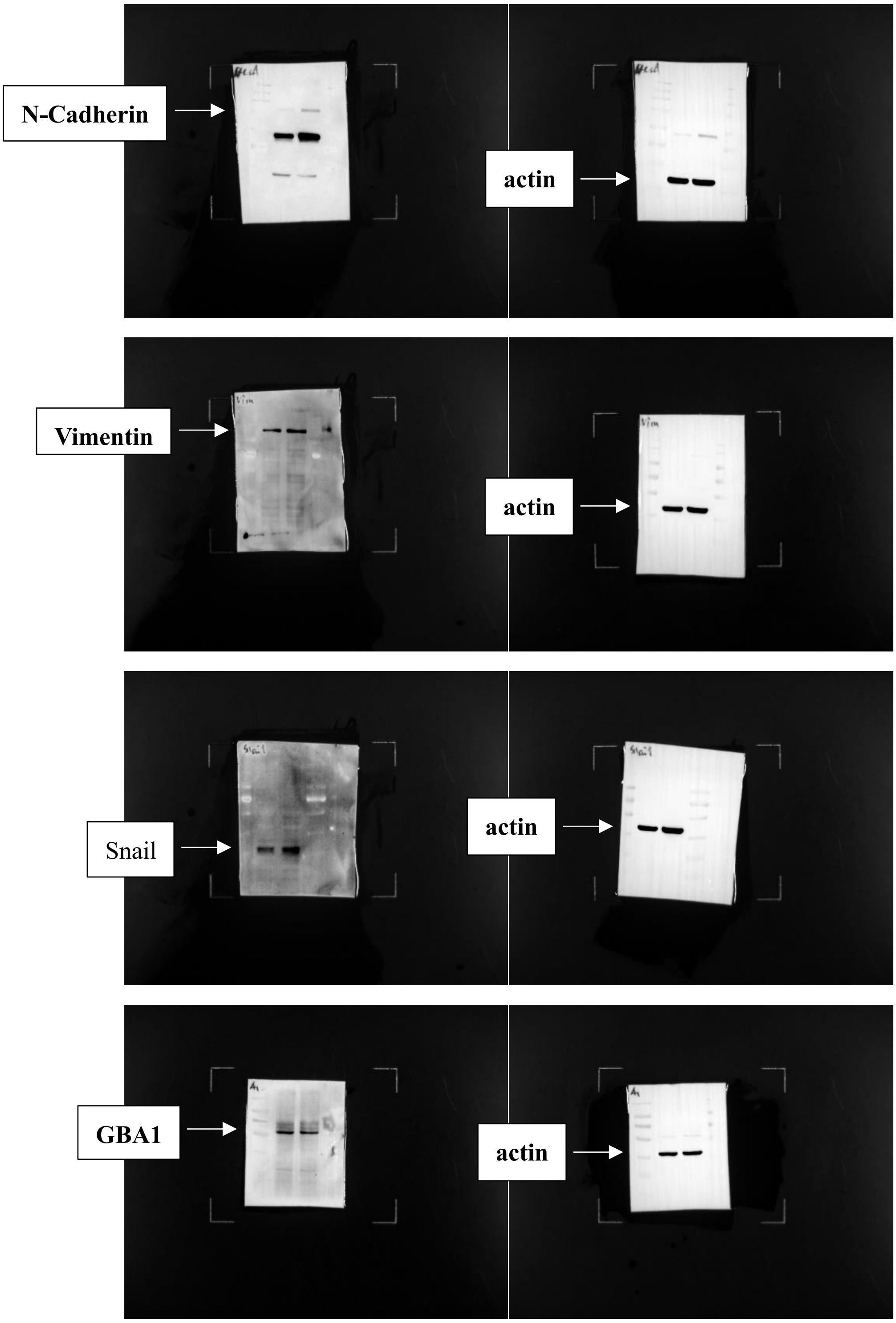

(B) The expression of phospho-LRP6 and the Wnt target genes c-myc and cyclin D1, which are related to the Wnt signalling pathway in MHCC-97H cells with stable GBA1 overexpression.

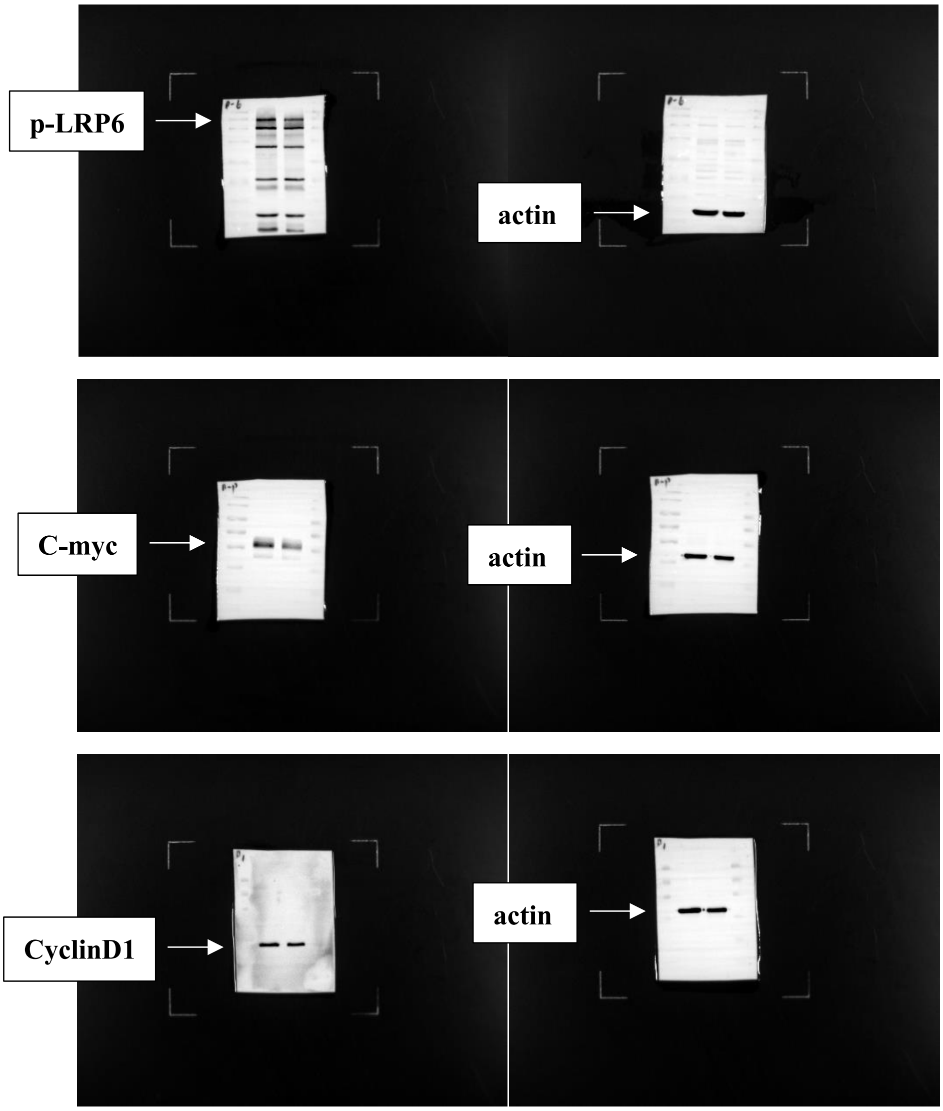

(E) The expression of phospho-LRP6 and the Wnt target genes cyclin D1, which are related to the Wnt signalling pathway HepG2 cells with stable GBA1 downregulation.

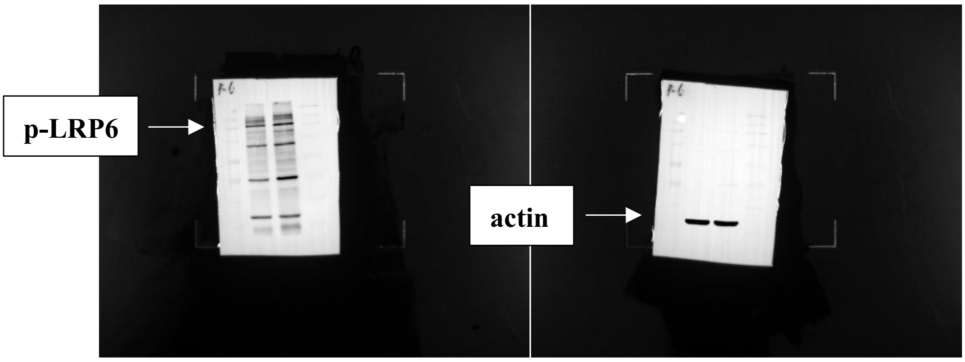

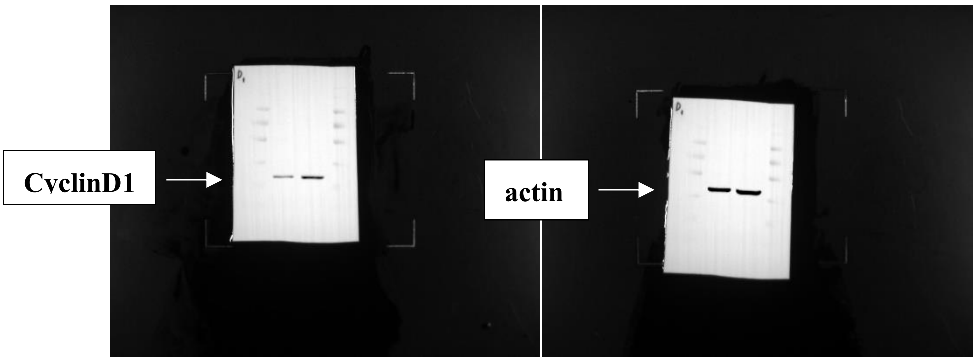

(C) Western blot analysis was performed to determine the level of non-phospho-β-catenin in the cytoplasm and nucleus of MHCC-97H cells with stable GBA1 overexpression

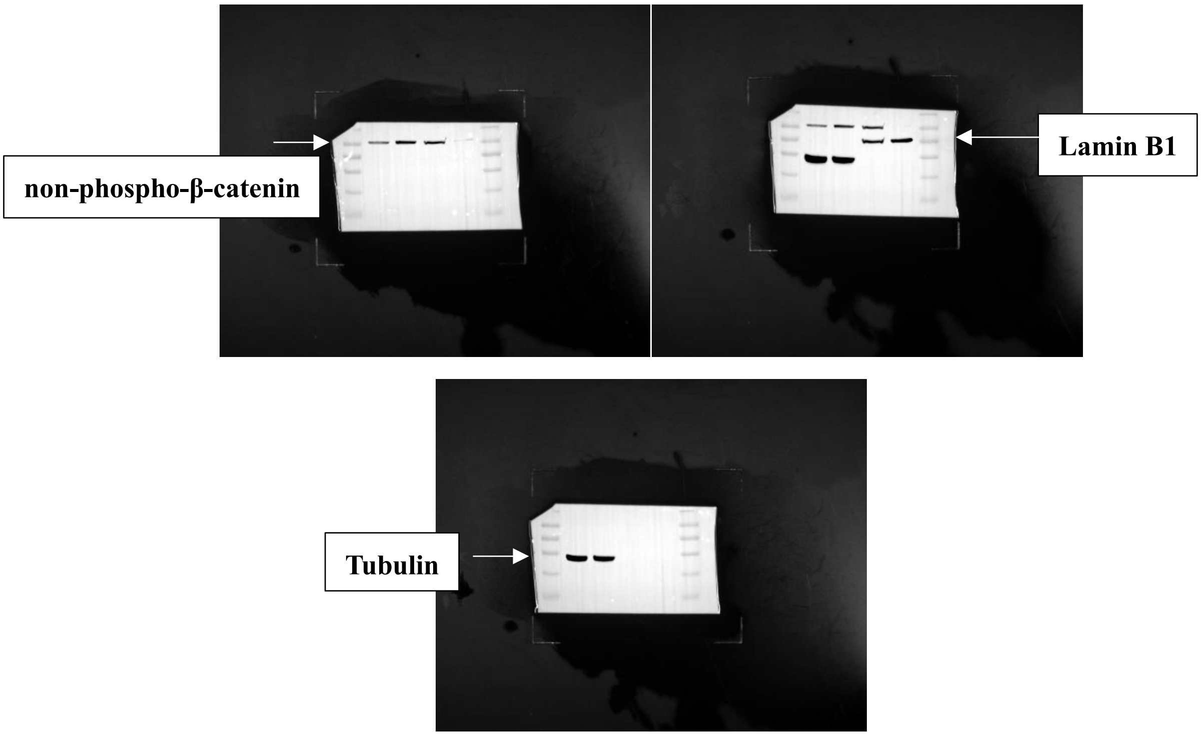

(F) Western blot analysis was performed to determine the level of non-phospho-β-catenin in the cytoplasm and nucleus of HepG2 cells with stable GBA1 downregulation.

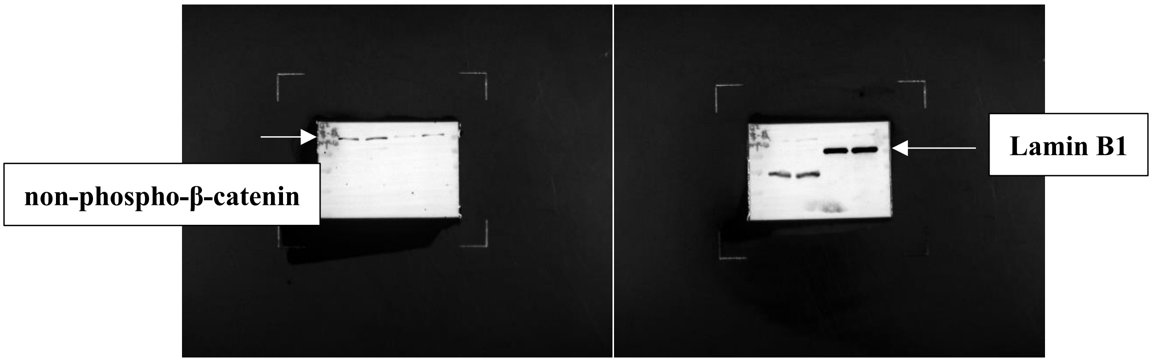

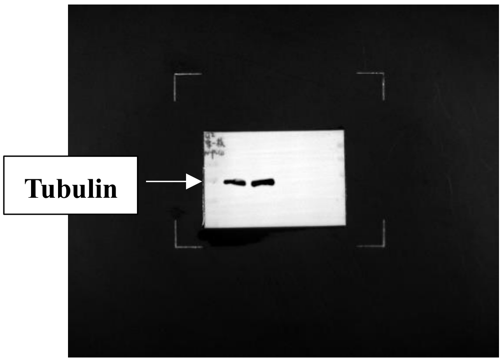

(G) Western blotting was performed to analyse the effect of GBA1 overexpression on the Wnt3a-activating EMT.

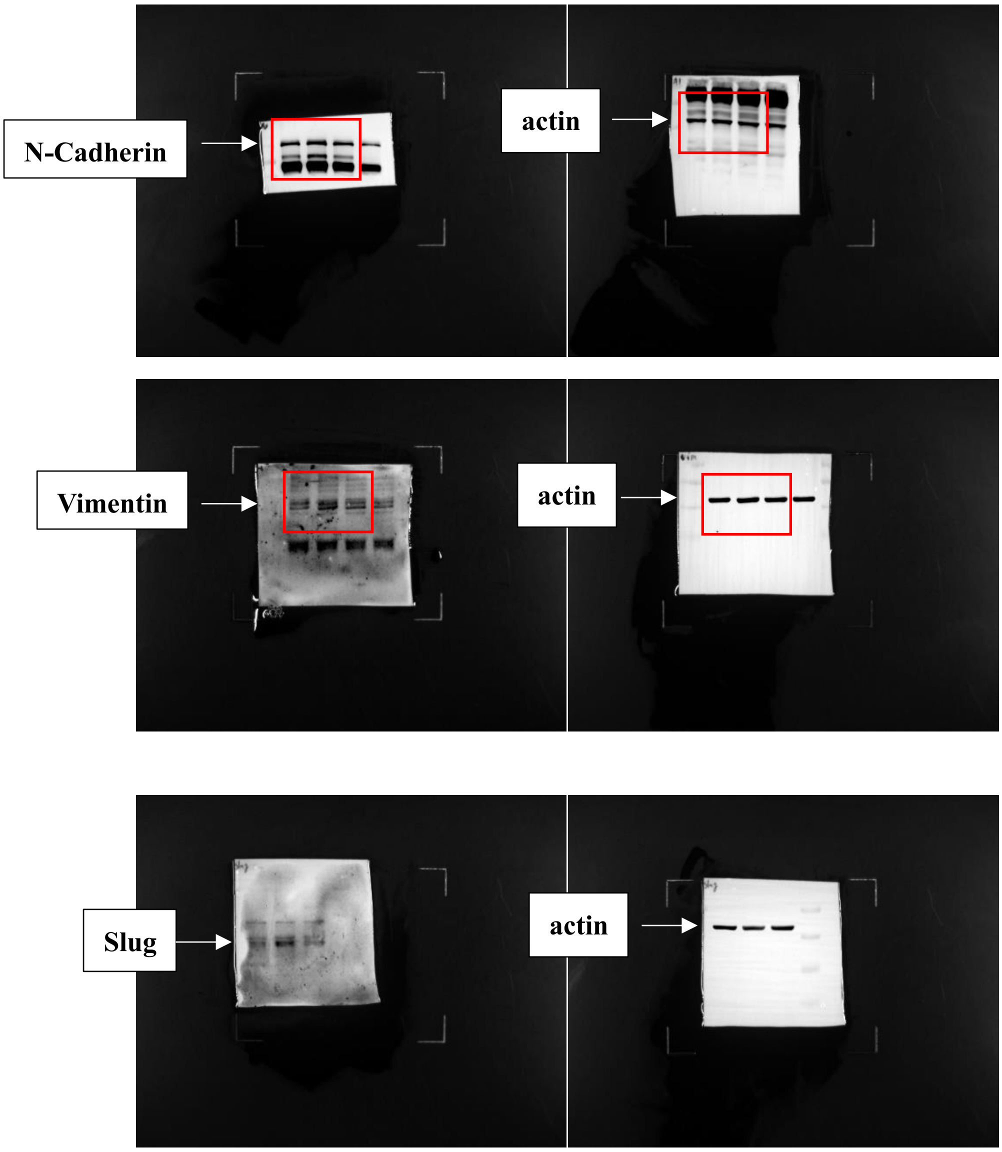

(H) Western blotting was performed to analyse the effect of GBA1 overexpression on the Wnt3a-

activated Wnt signalling pathway.

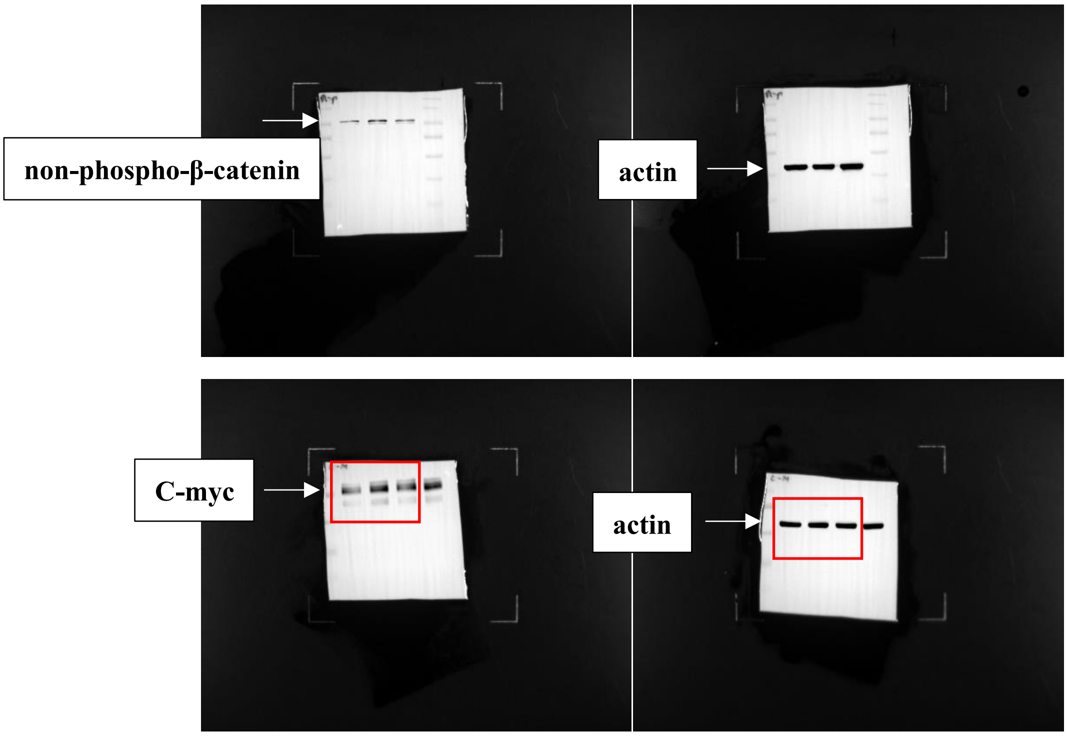

Figure 5 Low expression of GBA1 increases the levels of LRP6 in the cell plasma membrane and led to an interaction between GlcCer and LRP6

(B) Western blotting was performed to determine the levels of LRP6 in the cytoplasm and plasma membrane in HepG2 cells with stable GBA1 downregulation

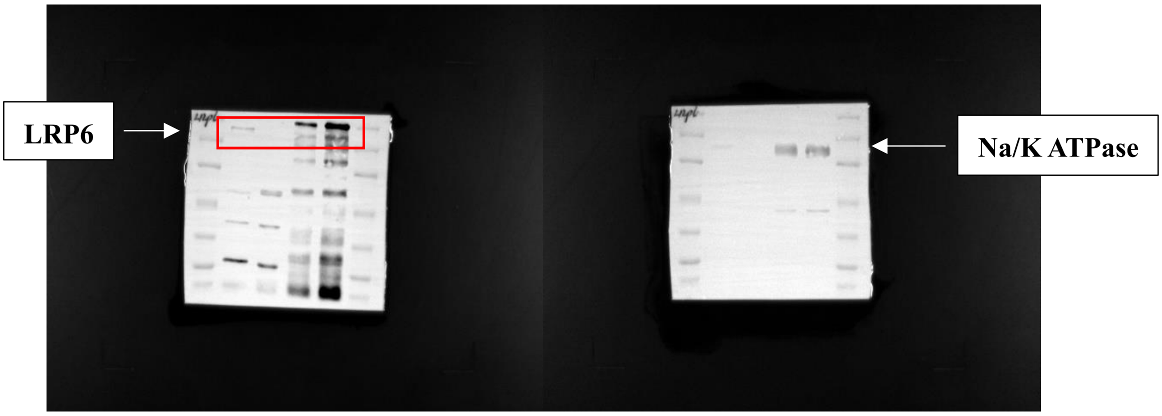

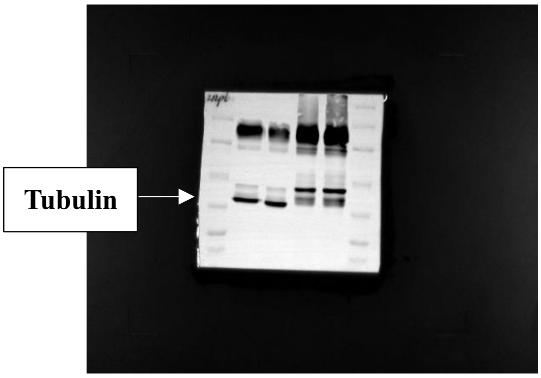

(C) Western blotting was performed to determine the levels of LRP6 in the cytoplasm and plasma membrane in MHCC-97H cells with stable GBA1 overexpression.

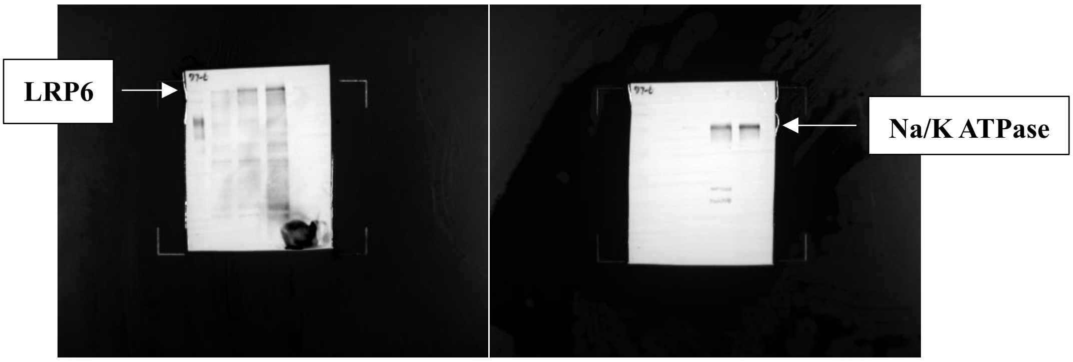

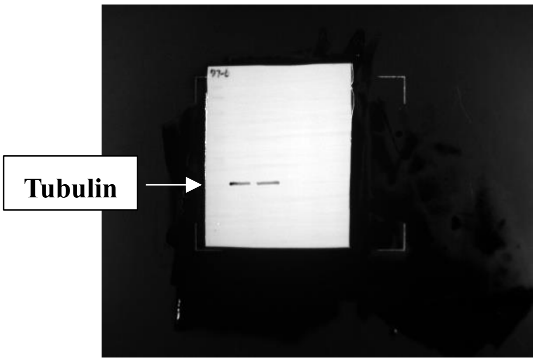

(E) Western blotting was performed for the phosphorylation of LRP6 at Ser1490 in HepG2 cells with stable GBA1 downregulation.

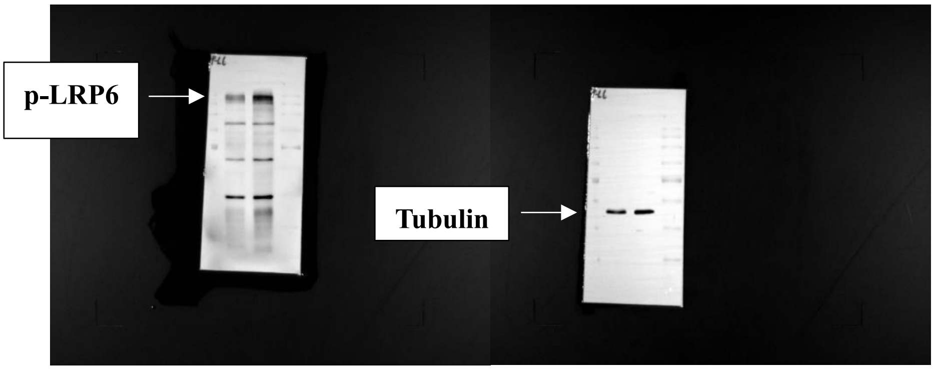

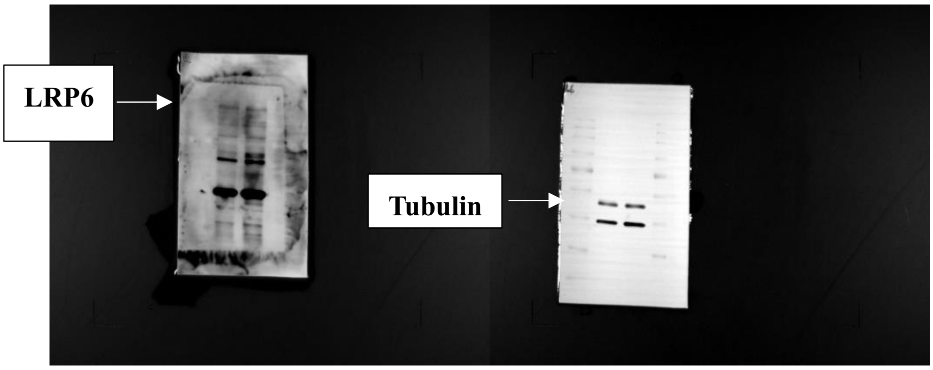

Figre 6 GlcCer plays a crucial role in promoting the metastasis of liver cancer cells

(C) Western blotting was performed to determine the effect of PDMP on GBA1 deficiency-upregulated proteins involved in the Wnt signalling pathway and EMT.

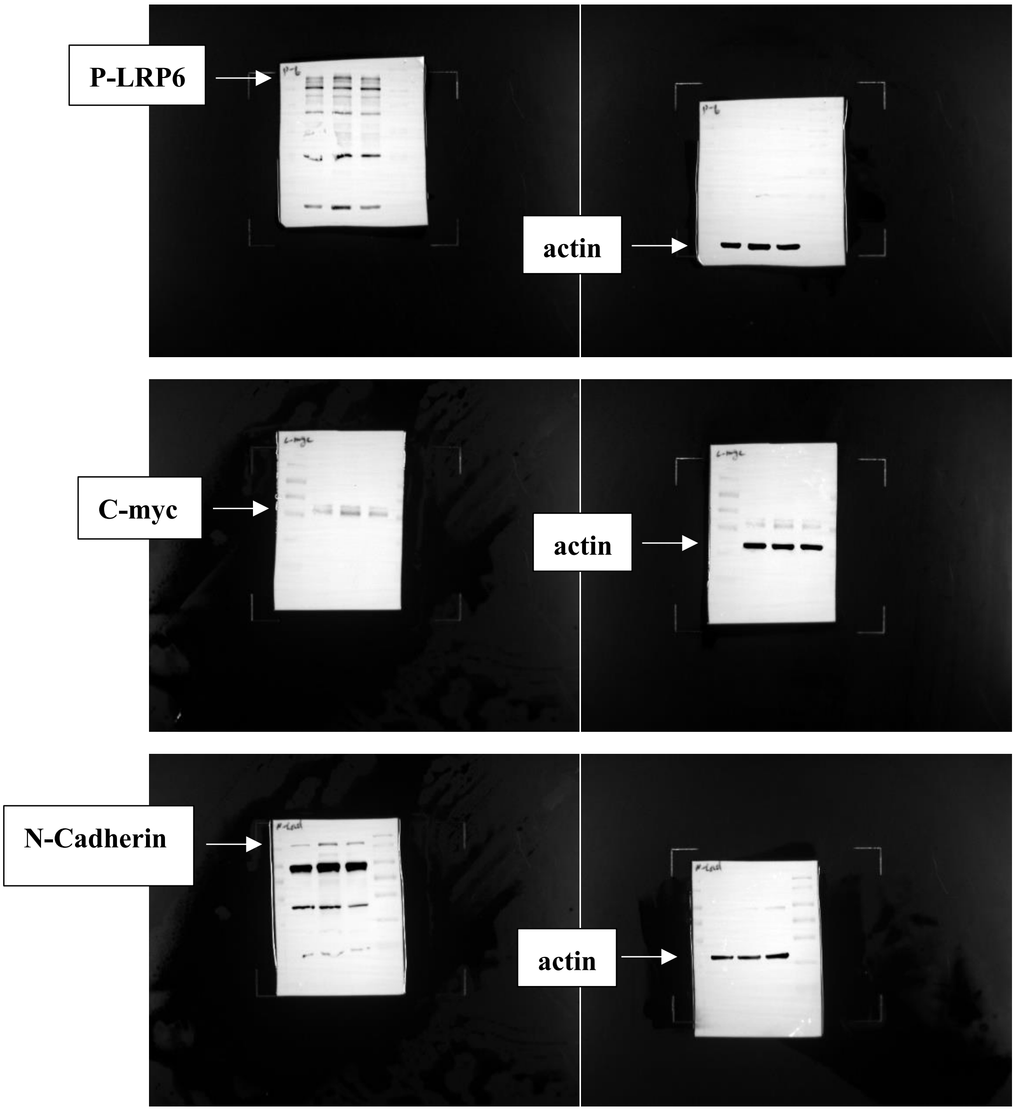

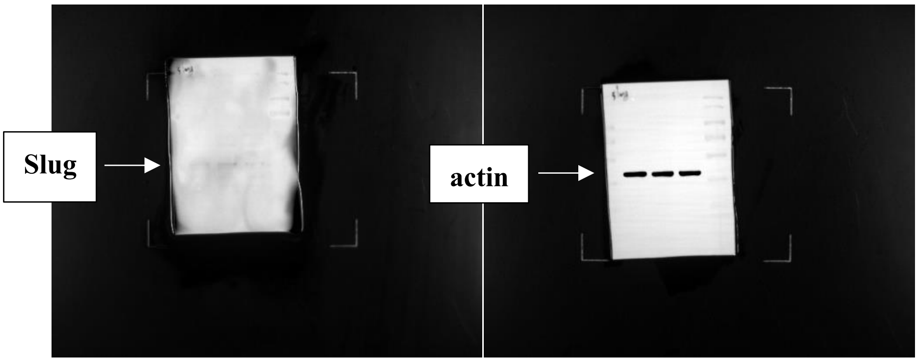

(E) Western blotting was performed to determine the effect of miglustat on GBA1 deficiency-upregulated proteins involved in the Wnt signalling pathway and EMT.

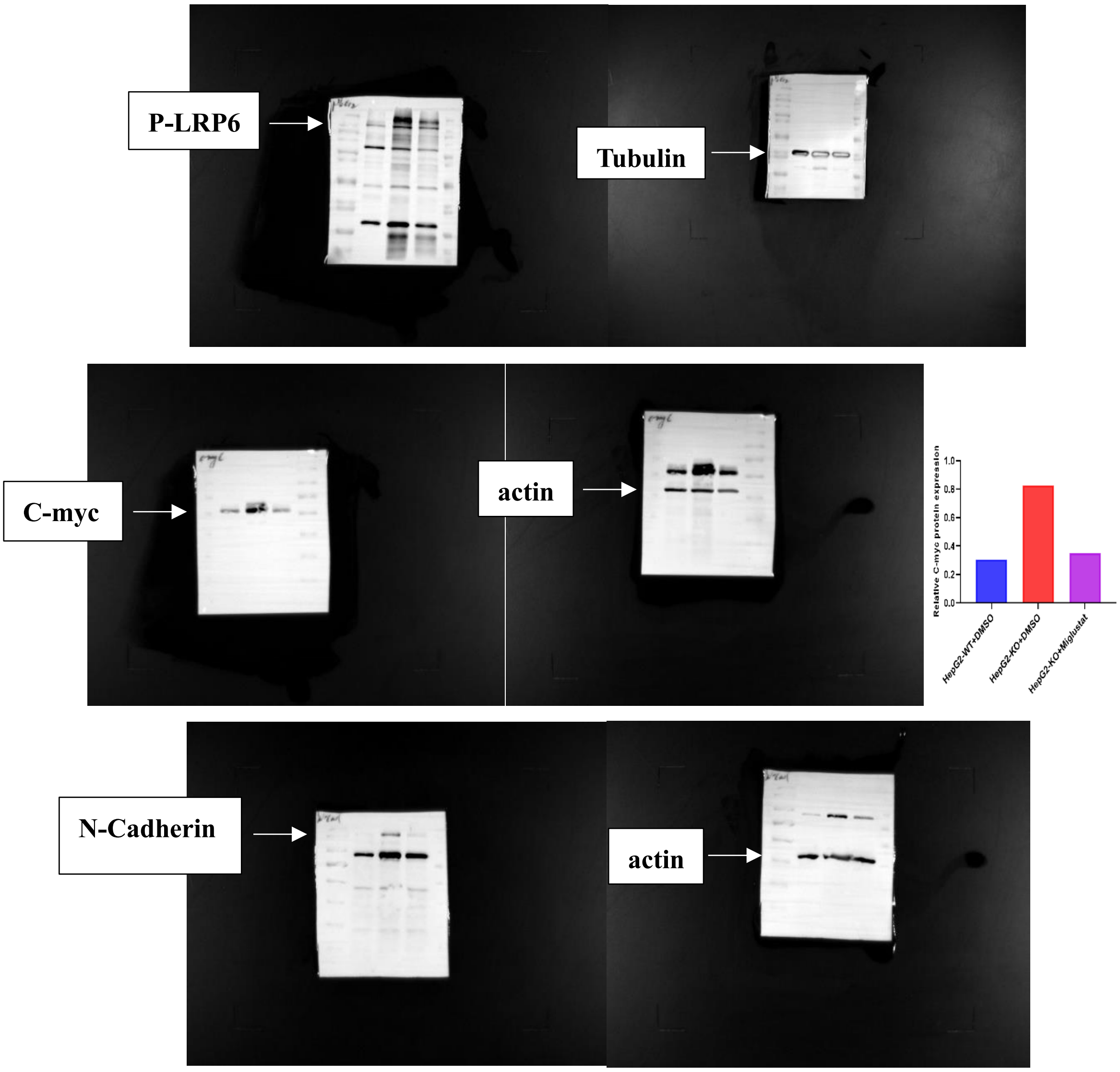

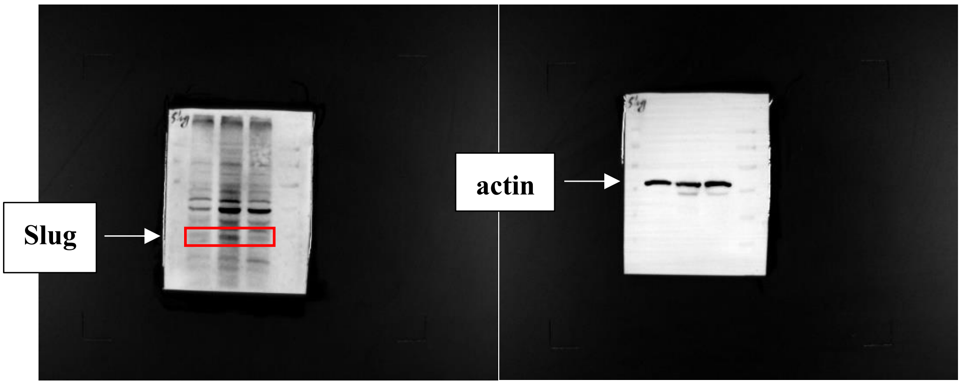

Figure 7 Downregulation of GBA1 is correlated with a high level of GlcCer, activation of the Wnt/β-catenin signalling pathway, and a high capacity for human liver cancer metastasis

(D) Western blotting was performed to determine the expression of Wnt signalling proteins and EMT markers in human liver cancers with metastatic lesions (MHs) and nonmetastatic lesions (NMHs).

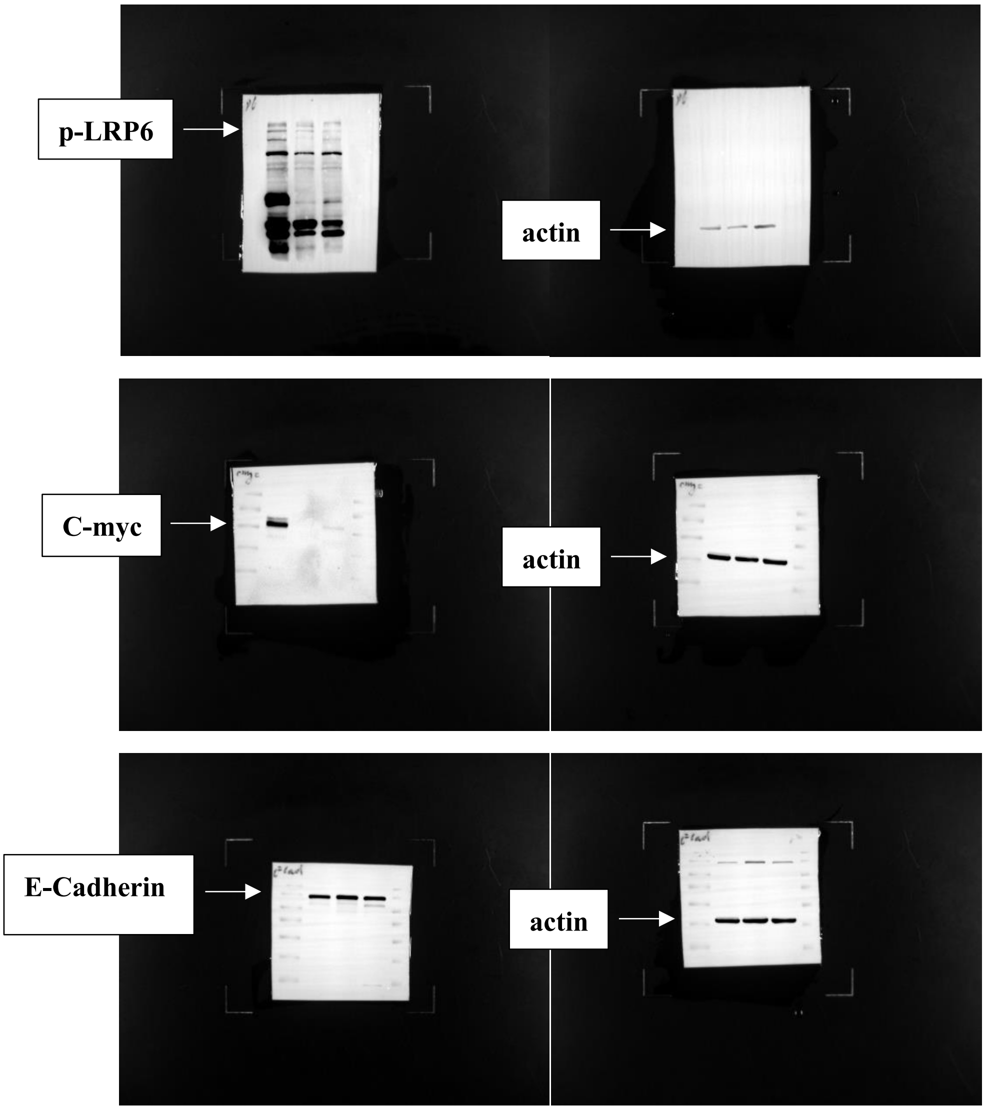

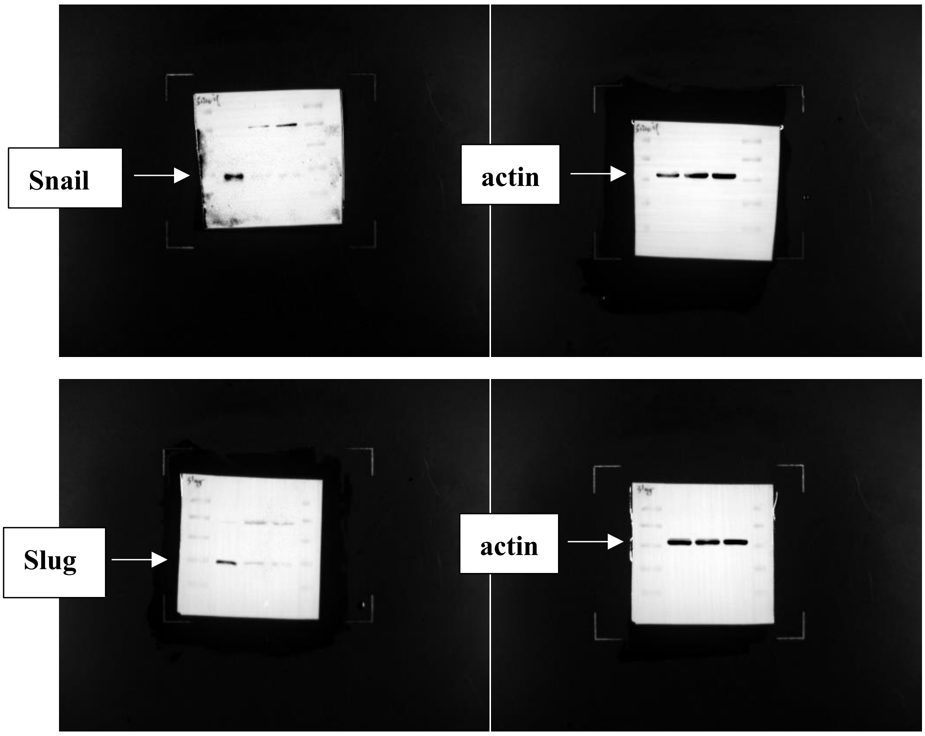

Figure S3 Upregulation of GBA1 reduced Wnt3a activating Wnt/β-catenin signalling pathway and EMT in the orthotopic xenograft model

A. Confirmation of Wnt3a overexpression in the MHCC-97H-Control and MHCC-97H-GBA1 stable cell lines.(Full-length Wnt3a (Myc-DDK tagged) was inserted into pLenti-C-mGFP vector)

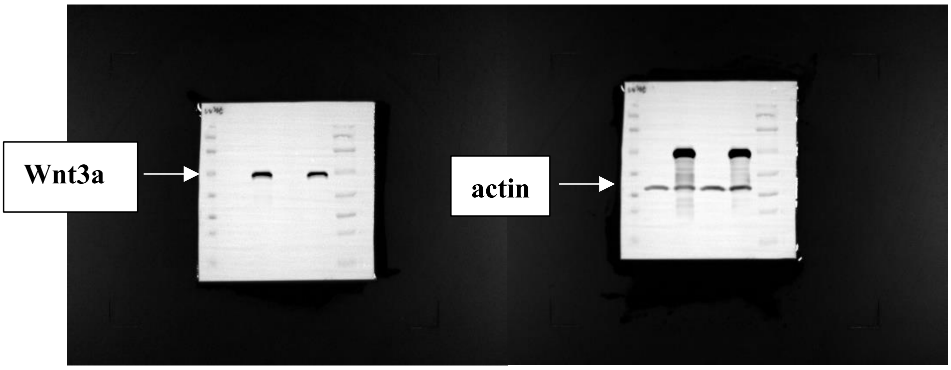

Figure S5 Low GBA1 inhibited sensitivity of sorafenib in HCC cells

(C) Differential protein expression levels of GBA1 siRNA knockdown and transient overexpression in Huh7s determined by Western blot.

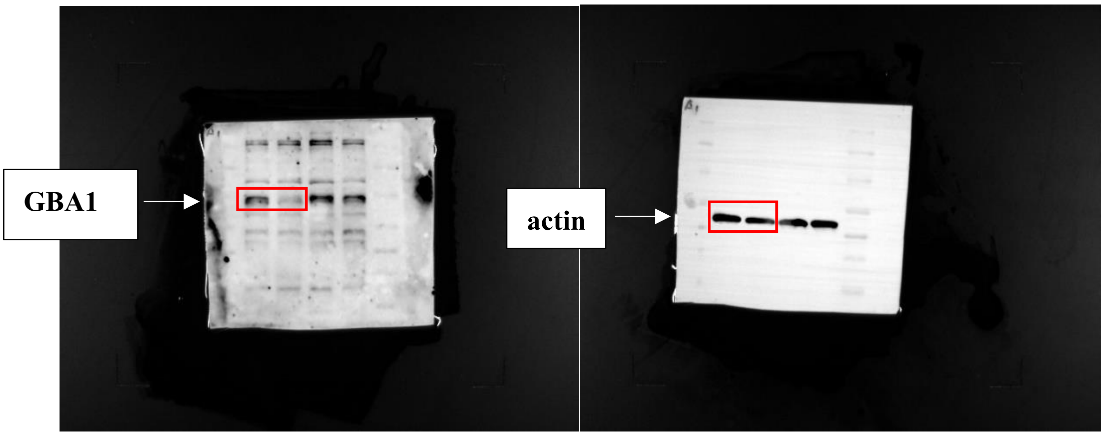
GBA1
actin

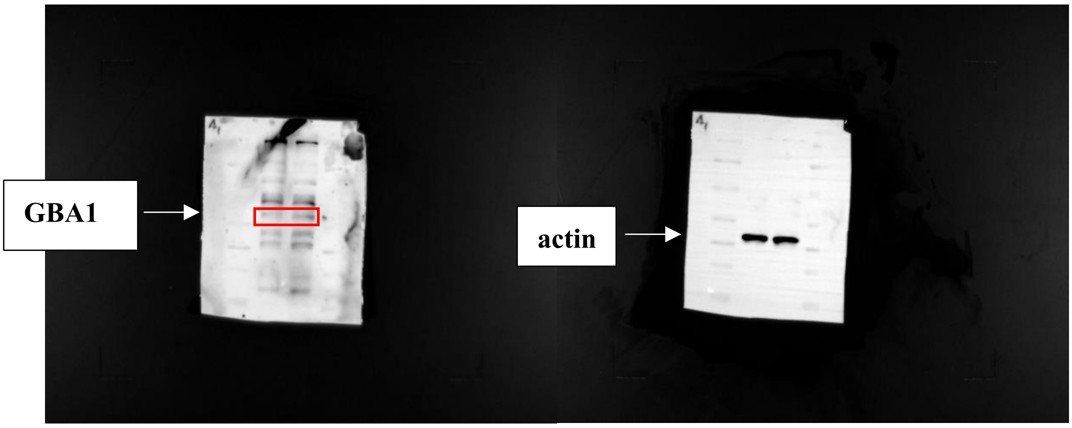
GBA1
actin
